# Transcription Factors Bind Thousands of Active and Inactive Regions in the *Drosophila* Blastoderm 

**DOI:** 10.1371/journal.pbio.0060027

**Published:** 2008-02-12

**Authors:** Xiao-yong Li, Stewart MacArthur, Richard Bourgon, David Nix, Daniel A Pollard, Venky N Iyer, Aaron Hechmer, Lisa Simirenko, Mark Stapleton, Cris L. Luengo Hendriks, Hou Cheng Chu, Nobuo Ogawa, William Inwood, Victor Sementchenko, Amy Beaton, Richard Weiszmann, Susan E Celniker, David W Knowles, Tom Gingeras, Terence P Speed, Michael B Eisen, Mark D Biggin

**Affiliations:** 1 Genomics Division, Lawrence Berkeley National Laboratory, Berkeley, California, United States of America; 2 Statistics Department, University of California Berkeley, Berkeley, California, United States of America; 3 Biophysics Graduate Group, University of California Berkeley, Berkeley, California, United States of America; 4 Department of Molecular and Cell Biology, University of California Berkeley, Berkeley, California, United States of America; 5 Life Sciences Division, Lawrence Berkeley National Laboratory, Berkeley, California, United States of America; 6 Affymetrix, Inc., Santa Clara, California, United States of America; 7 Center for Integrative Genomics, University of California Berkeley, Berkeley, California, United States of America; 8 California Institute for Quantitative Biosciences, Berkeley, California, United States of America; University of California San Diego, United States of America

## Abstract

Identifying the genomic regions bound by sequence-specific regulatory factors is central both to deciphering the complex DNA *cis*-regulatory code that controls transcription in metazoans and to determining the range of genes that shape animal morphogenesis. We used whole-genome tiling arrays to map sequences bound in Drosophila melanogaster embryos by the six maternal and gap transcription factors that initiate anterior–posterior patterning. We find that these sequence-specific DNA binding proteins bind with quantitatively different specificities to highly overlapping sets of several thousand genomic regions in blastoderm embryos. Specific high- and moderate-affinity in vitro recognition sequences for each factor are enriched in bound regions. This enrichment, however, is not sufficient to explain the pattern of binding in vivo and varies in a context-dependent manner, demonstrating that higher-order rules must govern targeting of transcription factors. The more highly bound regions include all of the over 40 well-characterized enhancers known to respond to these factors as well as several hundred putative new *cis-*regulatory modules clustered near developmental regulators and other genes with patterned expression at this stage of embryogenesis. The new targets include most of the microRNAs (miRNAs) transcribed in the blastoderm, as well as all major zygotically transcribed dorsal–ventral patterning genes, whose expression we show to be quantitatively modulated by anterior–posterior factors. In addition to these highly bound regions, there are several thousand regions that are reproducibly bound at lower levels. However, these poorly bound regions are, collectively, far more distant from genes transcribed in the blastoderm than highly bound regions; are preferentially found in protein-coding sequences; and are less conserved than highly bound regions. Together these observations suggest that many of these poorly bound regions are not involved in early-embryonic transcriptional regulation, and a significant proportion may be nonfunctional. Surprisingly, for five of the six factors, their recognition sites are not unambiguously more constrained evolutionarily than the immediate flanking DNA, even in more highly bound and presumably functional regions, indicating that comparative DNA sequence analysis is limited in its ability to identify functional transcription factor targets.

## Author Summary

One of the largest classes of regulatory proteins in animals, sequence-specific DNA binding transcription factors determine in which cells genes will be expressed and so control the development of an animal from a single cell to a morphologically complex adult. Understanding how this process is coordinated depends on knowing the number and types of genes that each transcription factor binds and regulates. Using immunoprecipitation of in vivo crosslinked chromatin coupled with DNA microarray hybridization (ChIP/chip), we have determined the genomic binding sites in early embryos of six transcription factors that play a crucial role in early development of the fruit fly Drosophila melanogaster. We find that these proteins bind to several thousand genomic regions that lie close to approximately half the protein coding genes. Although this is a much larger number of genes than these factors are generally thought to regulate, we go on to show that whereas the more highly bound genes generally look to be functional targets, many of the genes bound at lower levels do not appear to be regulated by these factors. Our conclusions differ from those of other groups who have not distinguished between different levels of DNA binding in vivo using similar assays and who have generally assumed that all detected binding is functional.

## Abstract 3

ChIP/chip analysis indicates that sequence-specific transcription factors bind to overlapping sets of thousands of genomic regions in *Drosophila* embryos, but most regions are bound at low levels and many may not be functional targets of these factors.

## Introduction

Deciphering the transcriptional information contained in the extensive *cis*-acting sequences that direct intricate patterns of gene expression in animals is a major challenge in biology. Animal genomes encode several hundred (e.g., *Drosophila*) to several thousand (e.g., human) transcription factors [1–3]. These proteins mediate transcription by binding in a sequence-specific manner to *cis*-regulatory modules (CRMs) found throughout the nonprotein coding portions of animal genomes (reviewed in [4–7]). The cells in which a CRM will activate or repress transcription of its target gene(s) are determined by the number, affinities, and arrangements of the transcription factor recognition sequences contained in the CRM, the expression patterns of the regulators that bind these sequences, and how the various factors interact. Animal sequence-specific regulators, however, generally recognize short, degenerate DNA sequences that occur frequently throughout the genome, and only a small subset of these predicted recognition sequences are thought to be functional targets of the transcription factor in vivo [4]. Because we do not yet understand the rules governing transcription factor binding or combinatorial interactions between factors, it is a major challenge to identify animal CRMs de novo or to predict how genes will be regulated based on their flanking DNA sequences alone.

Closely linked to this challenge is the problem of understanding the control of morphogenesis by developmental regulatory networks. Animals are composed of complex three-dimensional arrays of cells whose movements, shape changes, divisions, and patterns of determination and differentiation are coordinated by master regulatory genes, many of which encode transcription factors. If we knew the range of genes directly controlled by these regulators, it would greatly aid studies of how they coordinate the complex processes of morphogenesis.

To address these twin challenges, we have initiated an interdisciplinary analysis of the regulatory network controlling spatial patterning in the Drosophila melanogaster blastoderm embryo [8–11]. This network has been studied extensively, and we can be fairly confident that most of the major regulators have been identified [12,13]. Approximately 50 transcription factors are known to play a role in patterning the pregastrula embryo, forming a series of transcriptional cascades that regulate the formation of the anterior–posterior (A-P) and dorsal–ventral (D-V) axes. To decipher the combinatorial code by which transcription factors interact, it will be essential to have data for the great majority of factors in a system, and it should be possible to derive such comprehensive data for the early *Drosophila* network.

**Figure 1:**
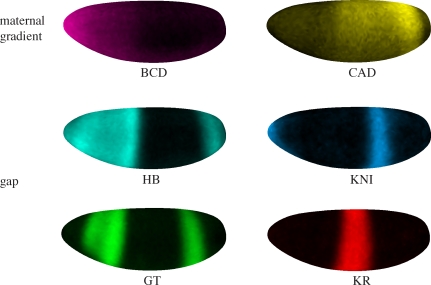
Patterns of mRNA Expression of the Six Maternal and Gap Genes Controlling Trunk Segmentation along the Anterior–Posterior Axis Expression (protein for BCD and mRNA for the other factors) is shown in orthographic projections using PointCloudXplore [100] to display data from the BDTNP's VirtualEmbryo [11] (BDTNP, unpublished data). The embryos are shown with anterior to the left, posterior right, dorsal at the top, and ventral at the bottom.

In this system, A-P patterning is initially established by maternally controlled activity gradients of two transcription factors: Bicoid (BCD), which has its highest activity in the anterior portion of the embryo and decays more posteriorly, and Caudal (CAD), which has its highest activity in the posterior portion of the embryo and decays anteriorly (Figure 1). Amongst the earliest zygotically transcribed genes are four targets of BCD and CAD—*hunchback* (*hb*), *Krüppel* (*Kr*), *knirps* (*kni*), and *giant* (*gt*)—the “gap” genes (Figure 1). These six genes encode transcription factors that work together to segment the A-P axis of the embryonic trunk (a collection of additional regulatory factors are involved in patterning the head and tail) [14–16]. For example, the second stripe of the pair-rule gene *even-skipped* (*eve*) is produced by the action of BCD, HB, KR, and GT on a CRM located approximately 1.5 kb upstream of the coding gene. In this case, BCD and HB act coordinately as activators in the same cells, whereas KR and GT each acts to repress expression in different parts of the embryo, restricting CRM output to a narrow stripe lying between the single band of KR expression and the anterior expression domain of GT [17,18]. This same collection of factors when bound to other CRMs produces different patterns of gene expression. The different combinations of recognition sequences in each CRM dictate the binding of factors in CRM-specific numbers and orientations. This binding, in turn, modulates the activity of each factor (in some cases changing activators into repressors, in others leading to binding-site competition or cooperative interactions) and produces a distinct transcriptional response [19–22]. Thus it is essential to study and model the action of these proteins in their native context.

In this paper, we use chromatin immunoprecipitation (ChIP) and Affymetrix whole-genome tiling arrays to map the genomic DNA regions bound by these six factors in D. melanogaster embryos. Our results provide the most comprehensive in vivo DNA binding data for a set of cooperating transregulators specifying complex spatial patterns of expression in an animal. They provide a framework for ongoing efforts to decode transcriptional information in the genome and model developmental regulatory networks.

## Results

### Genome-Wide Mapping of Bound Regions

To identify the genomic regions bound in vivo by the gap and maternal factors controlling trunk segmentation, we adapted chromatin immunoprecipitation and microarray (ChIP/chip) methods [23,24]. Briefly, intact blastoderm embryos (late stage 4 through stage 5) were treated with formaldehyde to crosslink proteins and DNA, after which chromatin was isolated, fragmented to an average length of 600 bp, and immunoprecipitated with antibodies recognizing the target protein [25]. The recovered material was amplified and hybridized to an Affymetrix whole-genome tiling array that contains over three million features representing 25-bp sequences spaced on average 35 bp apart across the unique portion of the D. melanogaster genome [26].

Our ChIP and DNA amplification protocols were optimized to maximize the signal-to-noise ratio, something that is especially critical in this system because these factors are only expressed at high levels in approximately 20% to 30% of cells (Figure 1). We also developed and optimized computational and statistical methods to provide an extensive, and accurate, high-resolution map of regions bound by each factor.

Data were obtained using affinity-purified antibodies to KNI, KR, HB, GT, BCD, and CAD. In addition, to detect genes that are transcribed at this stage of development, further immunoprecipitations were performed using a monoclonal antibody recognizing the phosphorylated form of the C-terminal heptapeptide repeat of RNA polymerase II [27].

To reduce the possibility that the antibodies against gap and maternal factors might cross-react with proteins other than the one against which they were raised, we affinity purified all antisera against recombinant proteins engineered to remove amino acid sequences found in any other *Drosophila* proteins. For BCD, HB, KR, and KNI, we used two different antibody preparations that were independently purified against nonoverlapping epitopes; for CAD and GT, we were only able to obtain one set of purified antibodies per protein.

**Figure 2:**
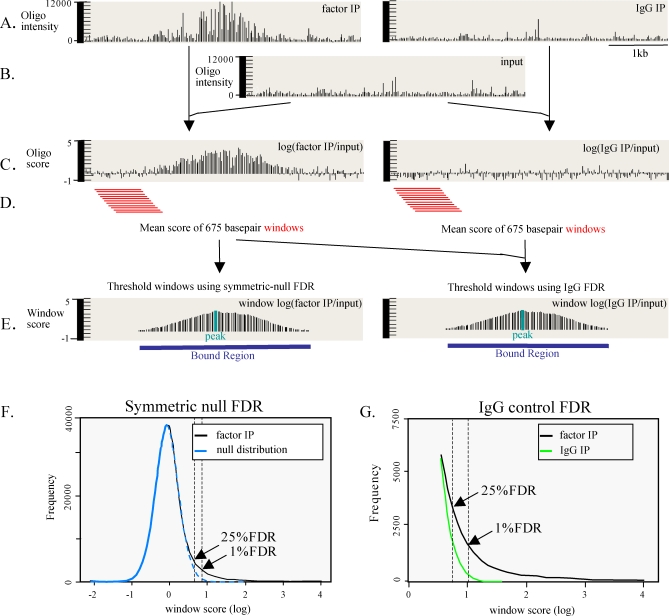
Overview of ChIP/chip Data Analysis Methods (A–C) Mean hybridization intensities (A) for Factor IP replicates (left) and IgG control IP replicates (IgG) (right) are divided by the mean probe intensity in the input DNA samples (B) to produce oligonucleotide ratio values (C). (D–G) The logarithms of the ratios in (C) are averaged in windows (D) of 675 bp centered around each probe (after discarding the highest and lowest values, to produce a “trimmed mean”) to produce window scores (E). Bound regions (E) were identified by comparing window scores to expected score distributions computed from a symmetric null distribution (F) or from IgG controls (G). The symmetric null method assumes that the background window score distribution is symmetric about its mean, and estimates the distribution from values less than the observed mode ([F] light-blue line). This estimated null distribution was used to assign *p*-values to each window score, and these were corrected for multiple testing to control the FDR, using the method of ([95] and http://faculty.washington.edu/∼jstorey/qvalue/). The IgG control is similar except that the empirical distribution of window scores from the IgG immunoprecipitations ([G] light-green line) is used as the estimated null distribution.

For each purified antisera, two independent replicates of three different sample types were analyzed on separate arrays: (1) “Factor immunoprecipitates (IPs)” obtained by immunoprecipitation using a factor-specific antibody; (2) “immunoglobulin G (IgG) control IPs” obtained by immunoprecipitation using a normal IgG antibody; and (3) “input DNA” obtained from the chromatin prior to immunoprecipitation, for a total of six arrays per antibody (Figure 2A and 2B).

To correct for the nonuniform hybridization response of the 25-bp oligonucleotides [28,29], we divided the mean hybridization signal for each array element in the Factor IPs and IgG control IPs by the mean hybridization signal for the same feature in the input DNA (Figure 2C). To further reduce noise, logarithms of these calculated oligonucleotide ratio scores were averaged (throwing out the highest and lowest values to produce a “trimmed mean”) in 675-bp windows (approximately equal to the size of the immunoprecipitated fragments) centered around each array element to give a hybridization window score (Figure 2D).

To determine which window scores represent significant enrichment in the Factor IP samples, we estimated false-discovery rates (FDR; the fraction of windows with equal or greater scores that are not detectable enriched in the IP) using two separate methods (Figure 2F and 2G). One method determined empirically the distribution of scores for unenriched windows using the distribution of window scores in the IgG control IP (Figure 2G; Materials and Methods); the other method estimated this distribution directly from the Factor IP using a “symmetric null distribution” method [30] (Figure 2F).

For each FDR estimation method, all overlapping windows with mean hybridization scores whose corresponding FDRs were less than either 0.01 or 0.25 were collapsed into contiguous bound regions. Each bound region was assigned a hybridization score and FDR level equal to those of its highest scoring window, and the location of the maximum array hybridization within each bound region was determined and defined as its “primary peak window” (Figure 2E).

Our subsequent analyses focus on bound regions with FDRs below 0.01 and 0.25 (the 1% and 25% sets, respectively). On average, the 25% FDR sets contained three to six times the number of genomic regions as their respective 1% FDR sets. Table 1 summarizes the number of bound regions identified for each factor; Tables S1 and S2 provide lists of the regions bound by each factor, and the locations of primary peak windows, as well as information on genes proximal to the regions for the 1% FDR and 25% FDR sets, respectively.

**Table 1:**
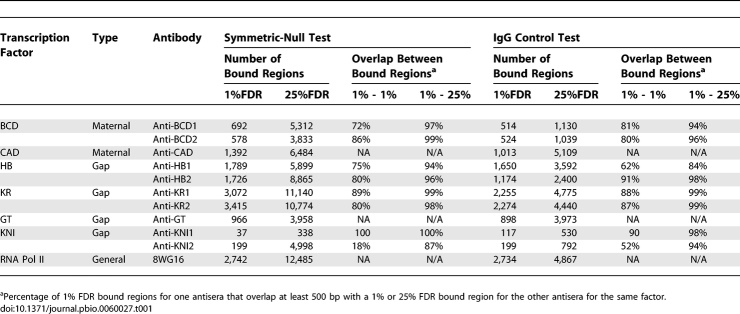
Number of Regions Bound by Transcription Factors

### Data Quality

**Figure 3:**
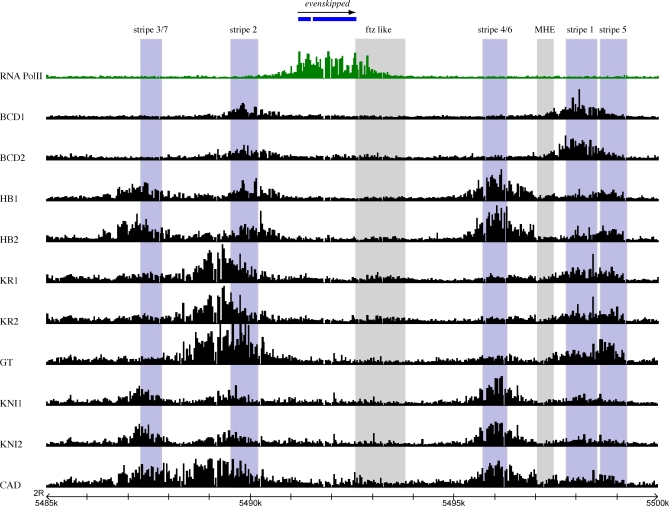
In Vivo Binding to the *even skipped* Locus Oligonucleotide ratio scores for all ChIP/chip experiments across the well-characterized *even skipped* locus. Data are shown for RNA PolII and all six factors. Note the agreement of the independently purified antibody for BCD, HB, KR, and KNI. The light-blue boxes mark the positions of experimentally characterized A-P enhancers regulating stripe 1 [21], stripe 2 [18], stripes 3 and 7 [101], stripes 4 and 6 [21], and stripe 5 [21]. For comparison, the grey boxes mark the positions of two enhancers that do not respond to these factors in the blastoderm, the *ftz*-like enhancer [21] and the muscle and heart enhancer (MHE) [102].

There is excellent agreement between technical replicates as well as data from immunoprecipitation experiments using antibodies recognizing distinct epitopes on the same transcription factor (see Figure 3). There is also good correspondence of the bound regions identified for the different antibodies (Table 1). On average, 96% of the 1% FDR bound regions detected with one antibody overlap regions that score above the 25% FDR threshold for the second (Table 1) (1% FDR regions were compared to 25% FDR regions to avoid not counting regions that lay just above the 1% FDR threshold for one antibody and just below for the other, which results in a somewhat lower overlap between the 1% FDR regions for both antibodies [Table 1]). Scatter plot analyses, presented later, also show a strong quantitative correlation between data from experiments using different antibodies to the same factor.

The two methods for estimating FDRs also broadly agree, especially at the 1% FDR level (Table 1 and Materials and Methods). (For simplicity, in the remainder of the paper, we use only the symmetric null FDR estimates.)

To confirm the accuracy of our FDR estimates, we randomly selected 33 regions from the KR 1% FDR set and 23 regions from the BCD 1% FDR set, and amplified approximately 100-bp fragments close to the hybridization intensity peaks in each region by quantitative polymerase chain reaction (Q-PCR) from immunoprecipitated DNA. All of the regions tested were enriched in immunoprecipitations using both of the independently affinity-purified antibodies used for BCD and KR (Figure S1). In addition, 11 out of 16 KR bound regions selected from the bottom half of the 25% FDR list were enriched by Q-PCR from immunoprecipitated DNA (Figure S1), consistent with there being a significant fraction of bona fide bound regions between the 1% and 25% FDR thresholds.

In vitro experiments have previously shown that UV or formaldehyde crosslinking correlates with levels of transcription factor occupancy on DNA [25,31,32]. To determine whether our experimental processing of immunoprecipitated material was distorting the levels of crosslinking, we carried out a series of control experiments. First, we applied the same series of amplification, labeling, and hybridization steps used for immunoprecipitated DNA to a sample of genomic DNA to which D. melanogaster bacterial artificial chromosomes (BACs) were added at known concentrations, and compared the data to unspiked genomic DNA. The ratio of signal intensities at oligos found in the BACs are lower than expected from the concentration of spiked BACs, but this compression is essentially monotonic and preserves the relative ranking of bound regions (Figure S2B). Second, control Q-PCR experiments using immunoprecipitated chromatin samples also support the view that amplification and array hybridization do not profoundly distort relative DNA concentrations (Figure S2A). Third, the relative primary peak window scores for BCD on a small collection of highly, moderately, and poorly bound regions are in line with the relative levels of in vivo UV crosslinking determined by direct Southern blot analysis of immunoprecipitated DNA [31].

In addition, the BAC experiments suggest that the great majority of regions significantly enriched after immunoprecipitation of chromatin will be detected by our array assay. We produced a set of 1% FDR regions for the spiked BAC DNA, and found that 100% of the regions present at 10-fold excess, 94% of the regions at 3-fold excess, and 51% of regions at 2-fold excess were present in the 1% FDR set. Although these model experiments do not precisely replicate the situation in the ChIP/chip experiments, they suggest that our amplification, hybridization, and array analysis methods are quite sensitive and should result in very few false negatives among moderately and highly enriched regions. The high concurrence between independent data from separate antibodies to the same factor also argue strongly against a significant false-negative rate among the 1% FDR regions.

### Genome-Wide Binding Overview

The most striking feature of the genome-wide data is the large number of bound regions identified for each factor (see Table 1), with most factors having thousands of bound regions. A total of 10.8 Mb are covered by 1% FDR bound regions identified for one or more of these factors, and a total of 6.6 Mb are within 500 bp of a primary peak for at least one of the regions bound above the 1% FDR threshold. These numbers represent 9.1% and 5.6%, respectively, of the 118.4 Mb euchromatic portion of the Release 4 genome sequence. A total of 40.38 Mb are covered by 25% FDR bound regions, and 31.2 Mb are within 500 bp of a primary peak of a 25% FDR bound region.

**Figure 4:**
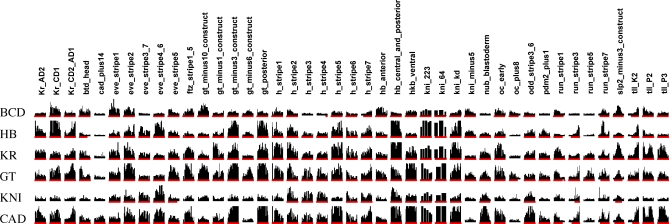
Known *cis*-Regulatory Modules Involved in Anterior–Posterior Patterning Are Highly Bound by Multiple Factors Oligonucleotide ratio scores for BCD1, HB1, KR1, GT, KNI2, and CAD for all *cis*-regulatory modules known to, or strongly believed to, be targeted by one or more of these factors prior to this study [8,9,33,103]. Only oligos within the region tested to demonstrate enhancer activity are shown. Red lines mark 1% FDR regions.

**Figure 5:**
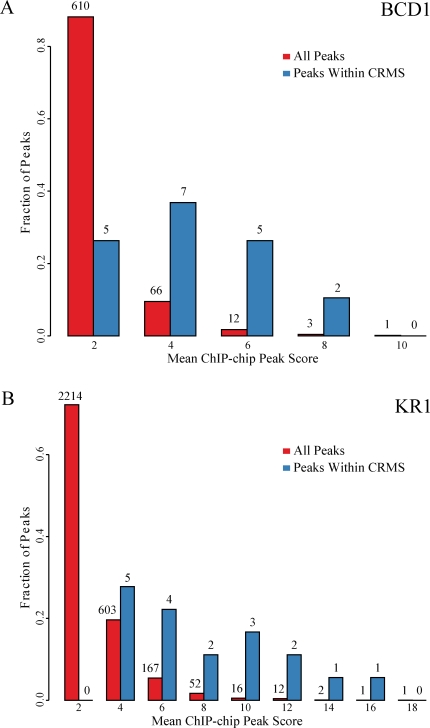
Known *cis*-Regulatory Modules Tend to Be among the Regions More Highly Bound In Vivo The 1% FDR bound regions for BCD1 (A) and KR1 (B) were each divided into cohorts based on primary peak window score (*x*-axis). The fraction of all bound regions in each cohort (red bars) and fraction of bound regions in each cohort in which the primary peak is contained within a CRM known to be regulated by the A-P factors (blue bars) are shown. The number of bound regions in each cohort is given above the bars.

These bound regions include all of the 43 CRMs known to be targets of one or more of these gap and maternal factors [9,33] (see Figures 3 and 4), arguing again that the false-negative rate in our ChIP/chip assay is very low. The known targets are, on average, more highly crosslinked than most of the identified bound regions, although some known targets are only poorly crosslinked (Figures 5 and S3), suggesting that lower levels of signal on the array may correspond to functional binding. In addition, the array hybridization signal frequently extends at low, but significant, levels many kilobases beyond the mapped edges of these known CRMs (e.g., Figure 3). Although part of this flanking signal close to the CRMs is due to hybridization of immunoprecipitated DNA fragments that include the CRMs, because the signal extends well beyond the length of the DNAs immunoprecipitated, much of it must be due to crosslinking of the factors to sequences outside of the mapped CRMs (for separate in vivo UV crosslinking data supporting this, see [32,34]).

There is considerable overlap of the regions bound by each factor. Collectively, 82% of 1% FDR bound regions overlap a 25% FDR bound region by at least 500 bp for at least one of the other five factors (Table 2).

**Table 2:**
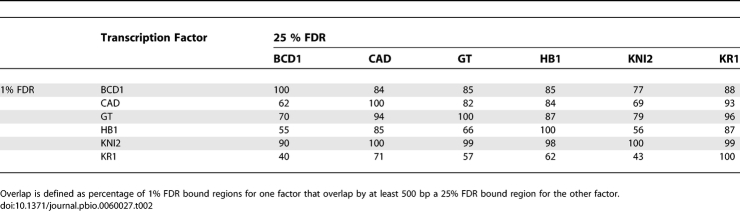
Overlap between Bound Regions for Different Factors

**Figure 6:**
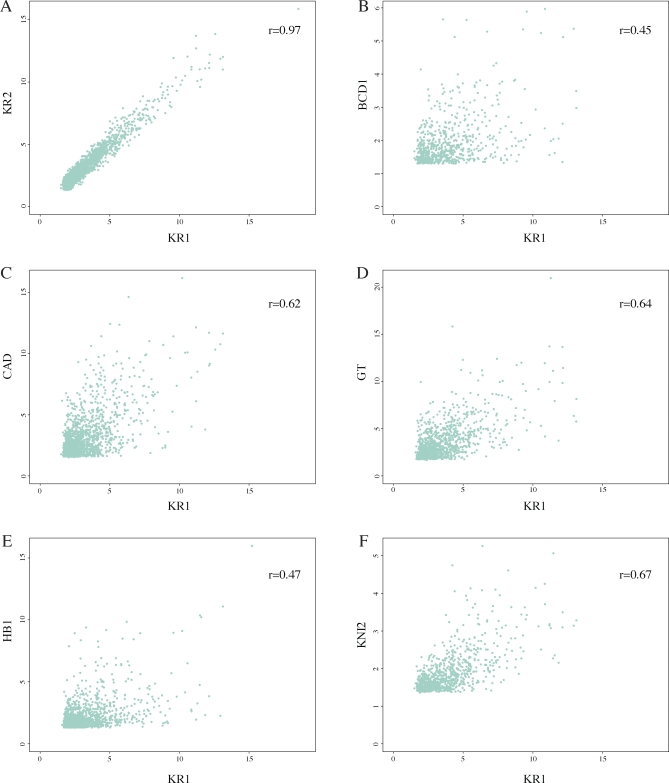
Factors Bind with Quantitatively Different Specificities to Shared Target Regions Correlation of primary peak score between overlapping (within 500 bp) primary peaks. (A) shows the correlation of KR1 1% FDR peaks versus KR2 25% FDR peaks; (B–F) show KR1 1% FDR against BCD1, CAD, GT, HB1, and KNI2 25% FDR primary peaks, respectively. The Pearson correlation coefficients (*r*) for each comparison are shown in the top right of each panel.

Despite this extensive overlap among the regions bound by each factor in vivo, each factor binds with quantitatively different preferences. For example, whereas there is strong correlation of hybridization intensities between different antibodies for KR, there is a lower correlation between the hybridization intensity of KR and the other factors in multiply bound regions (Figure 6). Similar results are seen for the other factors (Figure S4).

The variation in hybridization intensities for factors on the same target likely represents differences in the number of molecules of each factor occupying the shared target regions, either through differences in the number of recognition sequences bound and/or levels of occupancy at these sequences. Since many co-bound regions represent CRMs that direct different patterns of transcription, the quantitative differences in degree of binding of each factor must play a significant role in determining the unique output of these CRMs.

### Characteristics of Genomic Regions Associated with Bound Regions

Given the large number of in vivo binding regions identified in our analysis, the six gap and maternal factors may regulate a much broader array of genes and CRMs than the small collection of known target elements. To investigate to what extent the observed binding is associated with transcriptional regulation, we mapped each bound region to the gene transcribed in the blastoderm (based on our RNA polymerase II ChIP/chip data) whose 5′ end was closest to the center of bound region (see Tables S1 and S2). This mapping was imperfect due to the close packing of genes in the genome, the incomplete annotation of transcription units, and the ability of CRMs to act over large distances that sometimes skip intermediate genes; nonetheless, we still expected these associations to be broadly accurate.

**Figure 7:**
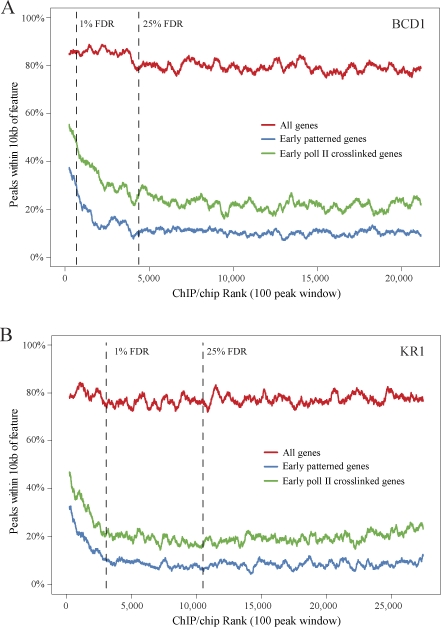
Highly Bound Regions Are Associated with Genes Transcribed and Patterned in the Blastoderm Analyzed are the percentage of primary peaks that are within 10 kb of the 5' end of a gene. Genes are divided into three categories, all genes (from genome release 4.3, March 2006), genes with known patterned expression (hand annotated based on Berkeley Drosophila Genome Project [BDGP] in situ images [35]) and transcribed genes (defined by our RNA Polymerase II ChIP/chip binding, see Materials and Methods). Percentages are calculated in nonoverlapping windows of 100 peaks down the rank list to the 80% FDR threshold. The position of the 1% and 25% FDR cutoffs are indicated with vertical dotted lines. (A) shows the results for the BCD1 antisera and (B) the results for the KR1 antisera.

The most highly bound regions for each factor were preferentially found near genes that are transcribed in the blastoderm (Figures 7 and S5). For example, 49% of the 100 regions most highly bound by KR are within 10 kb of a transcribed gene, but at the 1% FDR threshold, a point at which there are insufficient false positives to significantly affect the analysis, only 22% of regions are within that distance of a transcribed gene (Figure 7B). There is also a strongly nonrandom association of highly bound regions with genes that show patterned expression in the blastoderm (Figures 7A, 7B, and S5) [35].

**Figure 8:**
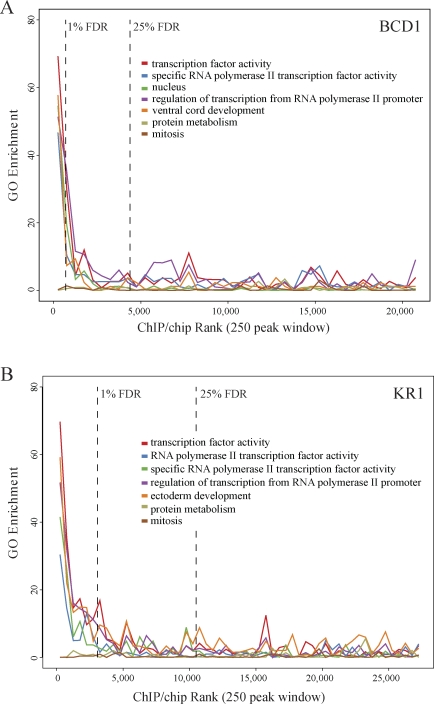
Genes That Control Development Are Highly Bound In Vivo The five most enriched GO ([98]) terms in the 1% FDR bound regions for each factor were identified (enrichment measured by a hypergeometric test). The significance of the enrichment (−log(*p*-value)) of these five terms plus those for two negative controls (protein metabolism and mitosis) in nonoverlapping windows of 250 peaks are shown down to the rank list for BCD1 (A) and KR1 (B) as far as the 80% FDR cutoff. The 1% and 25% FDR cutoffs are indicated by vertical dotted lines.

To further dissect these associations, we evaluated the enrichment of gene ontology (GO) terms of the putative targets of bound regions for each factor as a function of their position in the corresponding rank list (Figures 8 and S6). There is a consistent enrichment in the most highly bound regions for all factors of GO terms associated with A-P patterning, developmental control, and the regulation of RNA polymerase transcription.

**Figure 9:**
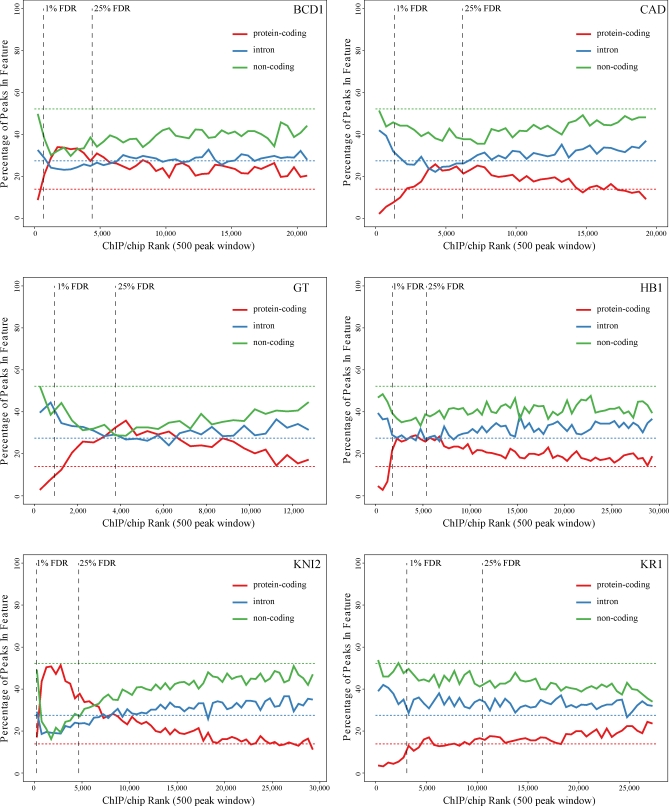
For Some Factors, Poorly Bound Regions Are Preferentially Found in Protein Coding Sequences Percentage of primary peaks in nonoverlapping windows of 500 peaks that are in protein coding (red), intronic (blue), and intergenic (green) sequence. Results are shown for windows down the rank lists to the 80% FDR cutoff. The percentages for each class of genomic feature are indicated as horizontal dotted lines in corresponding colors to the solid data lines. The 1% and 25% FDR cutoffs are indicated by vertical dotted lines. The panels show results for peak windows for BCD1, CAD, GT, HB1, KNI2, and KR1.

We also examined the percent of primary peak windows located in intergenic, intronic, 5′ and 3′ untranslated mRNA sequences, and protein coding regions (Figure 9). The great majority of the 500 most highly bound regions are found in intronic and intergenic sequences, as expected for transcription factor binding associated with gene regulation, but only at marginally higher frequencies than they would be found in a random selection of genomic regions (Figure 9). Surprisingly, for all factors except KR, many of the more poorly bound regions between the 1% and the 25% FDR thresholds are preferentially found in protein coding regions, which are not generally thought to code for CRMs (Figure 9).

Thus it appears that many of the most highly bound regions are involved in patterning nearby genes and the set of highly bound regions likely includes many new blastoderm CRMs. In contrast, many of the thousands of poorly bound regions seem unlikely to be acting as classical CRMs directing transcription in the early embryo. Some may instead be active as CRMs later in development, when they may be more highly bound by these same factors; others may have some as yet undetermined function distinct from transcriptional regulation. But it is quite possible that a substantial proportion have no function at all.

### Novel Targets Bound

**Figure 10:**
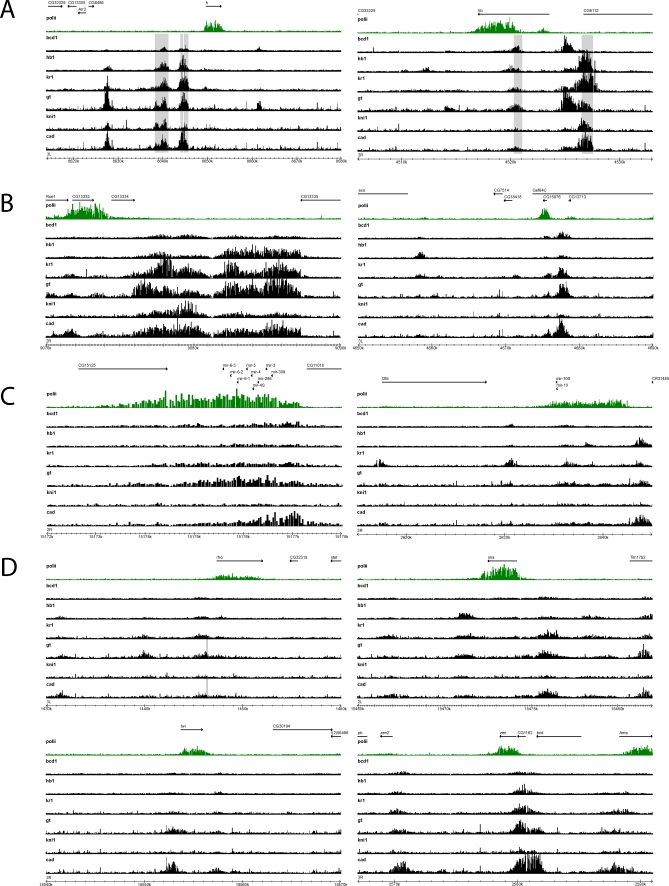
New Targets of Maternal and Gap Transcription Factors ChIP/chip oligonucleotide ratio scores for selected new targets. (A) Bound regions found near well-characterized A-P target genes *hb* and *h*, but which do not overlap known CRMs (shown in grey). For example, the binding 22 kb upstream of *h* is likely to be a novel *h* CRM because the other genes in proximity are not transcribed in the early embryo. (B) Genes transcribed in the early embryo that have no known function but are bound at moderate to high levels by multiple gap factors. In the left panel, *CG13333*/*CG13334* loci, and the right, *CG15876*/*CG13713* loci are shown. (C) miRNA genes that are actively transcribed in early embryos such as the gene that produces mir3, 4, 5, 6–1, 6–2, 6–3, 286, and 309 (left) and the mir-10 gene (right). See Table S3 for more details on binding to miRNA genes. (D) Binding in the region of D-V genes *rho*, *twi*, *zen*, and *sna*.

Among the most highly bound regions are four interesting classes of putative novel CRMs. (1) The noncoding regions flanking genes already known to be controlled by the early A-P regulators contain sequences bound highly by several of these factors that are distinct from their known CRMs (e.g., Figure 10A). (2) Many genes not previously known to be targets of the A-P regulators, but which are transcribed in spatial patterns in the blastoderm [35], are associated with regions bound by these factors (e.g., Figure 10B). (3) A large proportion of transcribed microRNA (miRNA) genes are flanked by regions bound by A-P regulators (Figure 10C, Table S3), consistent with the increasing evidence that miRNAs are spatially patterned and play important roles in developmental processes [36,37]. (4) Noncoding sequences near the principle zygotic regulators of the D-V axis are highly or moderately bound by many of the A-P regulators (e.g., Figure 10D).

**Figure 11:**
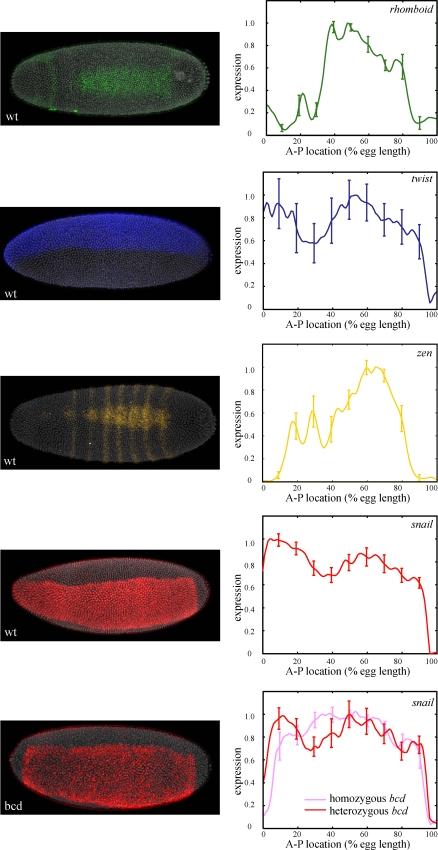
The mRNA Expression Patterns of Dorsal–Ventral Regulatory Factors Are Controlled by Anterior–Posterior Factors Data for wild-type embryos (top four rows) and embryos derived from *bcd* heterozygous and homozygous mothers (bottom row) are shown. Left panels show the mRNA expression patterns of representative embryos stained for *rho*, *twi*, *zen*, and *sna*. Dorsal views are shown for *sna* and *twi* and ventral views for *zen* and *rho*. Right panels show the mean mRNA expression for each gene along the A-P axis for a narrow strip of cells on either the dorsal or ventral midline. The error bars give the 95% confidence intervals for the means. The data for *rho* expression were derived from *n* = 22 wild-type (wt) embryos, *twist* from *n* = 14 wild-type embryos, *zen* from *n* = 10 wild-type embryos, and *sna* from *n* = 45 wild-type embryos, *n* = 24 embryos derived from *bcd* heterozygous mothers, and *n* = 24 embryos derived from *bcd* homozygous mothers.

Whereas the first three classes of putative target sequences are consistent with previous gene expression analysis, the observation that D-V regulators are bound was surprising as it has long been thought that they are not controlled by early A-P regulators. To test whether this binding might be functional, and whether previous regulation of D-V genes by A-P factors has been overlooked, we measured the mean levels of mRNA expression of four D-V regulators along the A-P axis using high-resolution imaging methods developed by the Berkeley Drosophila Transcription Network Project (BDTNP) [10,11]. Figure 11 shows that mRNA levels of the D/V regulators *rhomboid* (*rho*), *zerknult* (*zen*), *twist* (*twi*), and *snail* (*sna*) change significantly along the A-P axis in wild-type embryos. The larger changes in expression can be readily seen in images of individual stained embryos, but smaller or more gradual changes—that can be as large as 40%—are only reliably detected by quantitative analysis (Figure 11). In mutant embryos that lack BCD, the expression of *sna* mRNA along the A-P axis changes in the manner expected in these mutants because the posterior half of the pattern is duplicated as a mirror image in the anterior. The BCD mutant data and our binding data suggest that the early A-P regulators control expression of D-V genes. This result parallels similar recent observations of the binding and regulation of A-P genes by D-V factors. The expression of many A-P genes is modulated up to 2-fold along the D-V axis [10,11] and analysis of genome-wide binding of the D-V factors Dorsal, Snail, Twist, Medea, and Zerknult show that these proteins bind to many A-P genes [38,39] (unpublished data).

### DNA Recognition Sequences in Bound Regions

One of our chief motivations for determining the sequences bound by transcription factors in vivo is to understand the molecular mechanisms that target factors to DNA. To begin this analysis, we examined the distribution of predicted recognition sequences for each factor in its bound regions and in regions where we do not detect it binding.

**Figure 12:**
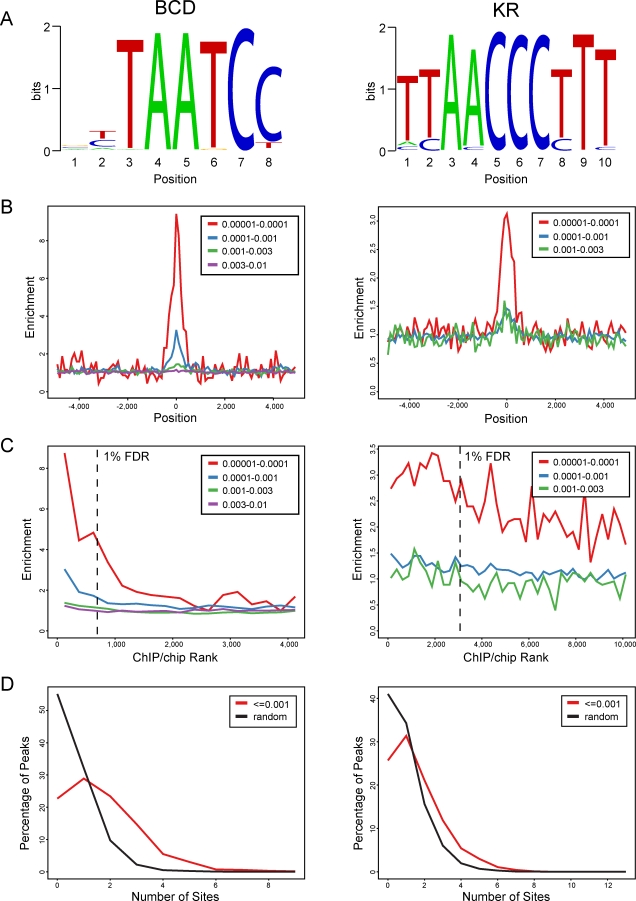
Recognition Sequences Are Modestly Enriched in Bound Regions (A) Sequence logo representing the BCD and KR PWMs derived from SELEX data (made with seqlogo [99]). (B) Fold enrichment of matches to the PWM from (A) in 100-bp nonoverlapping windows across the 1% FDR primary peaks, with the peaks at position zero on the *x*-axis. PWM matches shown are divided in subsets based on the *p*-value of their match to the matrix. (C) Fold enrichment of matches to the PWM from (A) in the 500 bp around (±250 bp) regions around primary peaks, in nonoverlapping windows of 250 peaks down the ChIP/chip rank list to the 25% FDR cutoff. The 1% FDR cutoff is indicated as a vertical dotted line. As in (B), matches are divided based on the significance of their match to the matrix. (D) shows the distribution of the number of sites in the 500-bp (±250 bp) regions around 1% FDR primary peaks and, for comparison, randomly selected noncoding genomic sequence. In this panel, a match to the matrix is defined as having a PATSER *p*-value of ≤0.001.

We derived position weight matrices (PWMs) for each of the six factors either from DNaseI footprint data of recognition sequences found in known enhancers [40] or from in vitro selection (SELEX) experiments (BDTNP, unpublished data). Two, BCD and KR, are shown in Figure 12A. Although such PWMs do not provide a complete description of a factor's binding specificity, they provide a first-order approximation [41].

We used these PWMs to identify all sequences across the genome that match each factor's in vitro binding specificity, and found that recognition sequences for each factor are enriched in their respective bound regions, the enrichment being greatest at the peak of array intensity hybridization (Figures 12B and S7B).

**Figure 13:**
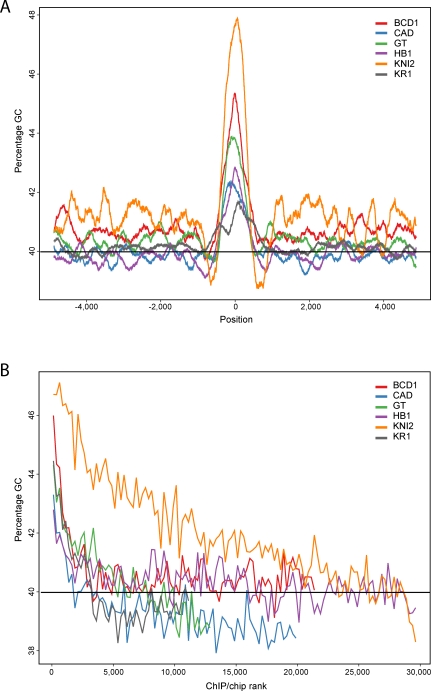
Bound Regions in Noncoding DNA Display a GC Bias (A) Average base composition (as represented by percentage GC) in nonoverlapping windows of 100 bp across the 10-kb regions (±5 kb) around 1% FDR primary peaks. (B) Base composition of the 500 bp (±250 bp) around primary peaks down the ChIP/chip rank list to the 80% FDR cutoff in nonoverlapping windows of 250 peaks. For both panels, only noncoding sequence was used in the analysis. The mean percentage GC content in noncoding DNA is indicated by the horizontal black line.

Such enrichment demonstrates that a significant fraction of the binding arises from the direct, sequence-specific interaction of each factor with its recognition sequences. However, bound regions, especially the most highly bound regions, show a marked G-C bias relative to their flanking sequences (Figure 13A). This bias could lead to the spurious observation of recognition sequence enrichment, especially because many transcription factors recognize G-C–rich sequences.

To ensure that the observed enrichment of recognition sequences for the gap and maternal factors in 1% FDR bound regions is not an artifact of general G-C bias, we repeated the enrichment analysis with PWMs generated by randomly permuting the order of columns within the real PWMs. Matches to these scrambled matrices are not enriched in bound regions, except in the case of HB whose homogenous PWM is not significantly altered by the permutation (Figure S9). However, a G-C bias would be expected to lead to a deficit of A/T-rich HB recognition sequences, and thus the enrichment of HB recognition sequences cannot be a result of G-C bias.

As a separate control, we examined the enrichment of recognition sequences for each factor in regions not bound by the factor, but bound by at least one of the other five. These regions are G-C rich, but again, only very modest or no enrichment is observed (Figure S10). Thus, the enrichment of recognition sequences is largely specific to regions bound by the factor and to the factor's correct PWM.

There is a strong positive correlation between the predicted affinity of a recognition sequence (estimated here by its score against a factor's PWM) and its enrichment (Figure 12B and S7B). For example, the eight highest-affinity variants of the 8-bp BCD recognition sequence (defined here as sequences that have log-likelihood scores against the BCD PWM of less than 0.0001; smaller log-likelihood values represent better matches to the PWM) are enriched over 8-fold in BCD bound regions relative to flanking noncoding DNA. In contrast, the 184 medium-affinity variants (those with log-likelihood scores between 0.001 and 0.003) are enriched only about 1.5 times over background. However, the total excess (compared to noncoding sequences in which we did not detect binding) of medium-affinity BCD recognition sequences in BCD bound regions, 1.3 recognition sequences per 1,000 bp of bound region, is higher than the excess of high-affinity recognition sequences, 1.0 per 1,000 bp (unpublished data), suggesting that medium-affinity sites likely play a significant role in targeting factors to DNA.

The enrichment of recognition sequences is greatest for the most highly bound regions, and declines with decreasing levels of in vivo binding (Figures 12C and S7C). Despite this decrease in enrichment, the high-affinity recognition sequences are enriched far down the rank lists.

Although recognition sequences are enriched on average in bound regions, consistent with previous data [32,34,42], the enrichment is modest and is not uniform among bound regions. A significant number of bound regions contain fewer recognition sequences for the bound factor than are found in many unbound regions (Figures 12D and S7D). For example, 80% of BCD 1% FDR primary peaks contain no predicted high-affinity BCD recognition sequences (*p* < 0.0001), and 20% do not contain any intermediate-affinity sequences (*p* < 0.001). Furthermore, there are numerous unbound regions that contain intermediate- and high-affinity recognition sequences.

### Excess of G-C Base Pairs in Bound Regions

The excess of G-C bias in bound regions noted above warranted further analysis. We were concerned that the correlation between strength of binding and G-C content might reflect a bias for G-C rich sequence to hybridize more strongly to the array. We used the BAC data described above to investigate the effect of G-C content on the hybridization score in the 675-bp windows we used in our analyses. There is a tendency for windows with (on average) low G-C content to have lower mean window scores (Figure S8), which could bias the selection of peak hybridization windows within bound regions towards those with higher G-C content. However, this effect would likely be somewhat countered by the tendency for windows with high G-C content to have lower mean window scores (Figure S8). In addition, peak window scores correlate with enrichment measured by Q-PCR, which is not subject to G-C bias (Figures S1 and S2). Finally, a similar GC bias has been observed in a large collection of enhancers not identified by array hybridization [43]. We therefore conclude that bound regions are G-C rich relative to other noncoding DNA.

### Recognition Sequences Enrichment Is Context Dependant

**Figure 14:**
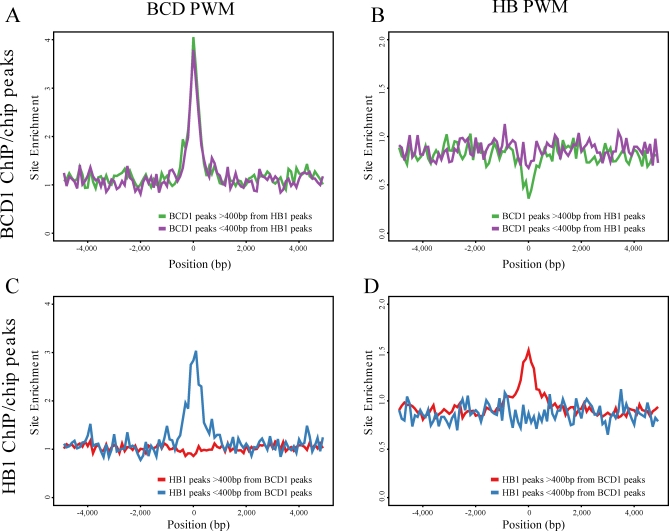
In Vivo Binding Is Influenced by a Context Determined by Other Factors (A) and (B) show the fold enrichment of BCD and HB PWMs in nonoverlapping windows of 100 bp across the 10-kb regions (±5 kb) around 1% FDR BCD1 peaks, divided into those less than and greater than 400 bp from HB 1% FDR peaks. (C) and (D) show the same analysis for 1% FDR HB1 peaks divided into those less than or greater than 400 bp from BCD1 1% FDR peaks. As (D) shows, HB recognition sequences are not enriched in regions bound by both HB and BCD, but are enriched in regions bound by HB and not by BCD.

Many lines of evidence suggest that animal transcription factors act in a context-dependant, combinatorial manner in which the action of one factor influences the behavior of another [44–49]. As a result, it is widely believed that a key to understanding how specific CRMs are constructed so that they are correctly bound by a defined set of transcription factors and produce specific patterns of expression lies in understanding a code that integrates information from multiple recognition sequences. For example, the ability to predict the locations of functional CRMs for the six early regulators is greatly improved when binding of multiple factors is considered at the same time, rather than when binding for factors is considered in isolation [8,9]. These observations suggest that the binding of one factor may influence the DNA binding or activity of other factors on an element. To begin to search for evidence of such effects in our dataset, we examined how the binding of additional factors influenced the frequencies of predicted recognition sequences for each factor in its bound regions (Figure 14). For some factors, such as BCD, little difference is seen, but for others. substantial, and in some cases counterintuitive, changes are seen. Predicted HB recognition sequences are enriched in regions bound exclusively by HB and by HB in combination with one or more of GT, KR, KNI, and CAD. However, predicted HB recognition sequences are not enriched in regions bound by HB and BCD (Figure 14D), even though these regions are crosslinked 1.3-fold more highly by HB than regions not bound by BCD (unpublished data). Thus there appears to be a complex influence of other factors on the manner in which at least HB recognizes its target sequences.

### Evolutionary Conservation of DNA Recognition Sequences

A critical question unanswered by the above analyses is what fraction of the regions bound in vivo are biologically functional, be they involved in transcriptional regulation or some other cellular function. Many of the most highly bound regions overlap CRMs known to regulate important developmental processes, and it is likely that many of the remaining highly bound regions, especially those near important developmental control genes, will have regulatory activity in the blastoderm. However, these regions represent just a small fraction of the regions bound by these six factors.

To begin to address the function of the remaining bound regions, we examined the evolutionary constraints on predicted recognition sequences in bound regions. We expect purifying selection to constrain substitutions at bases involved in protein–DNA interactions that mediate important regulatory events. Functional recognition sequences have consistently been observed to evolve more slowly than expected under neutral models [50], and evolutionary constraint on noncoding DNA is often used as a proxy for regulatory function (e.g., [39]).

The measurement and interpretation of constraint on recognition sequences, however, is not straightforward. First, *Drosophila* noncoding DNA is, in general, highly constrained. It has been estimated that over half of the bases in intergenic and intronic DNA have evolved under purifying selection [51,52], compared to roughly 5% in mammals [53]. Thus, when compared to the presumptive neutral rate of substitution (estimated in *Drosophila* from short introns), virtually any collection of recognition sites in *Drosophila* noncoding DNA will appear to be under evolutionary constraint, whether the sites are functionally bound or not.

Second, despite this generally high level of noncoding constraint, functional recognition sequences are not always conserved. For example, it has been shown that several functional recognition sequences from the *D. melanogaster eve* stripe 2 enhancer are absent in other *Drosophila* species even though the enhancers themselves maintain their function [54–56]. Presumably, the loss of these sites is compensated for by the gain of sites elsewhere in the enhancer.

Nonetheless, in most CRMs examined to date, at least a subset of recognition sequences are constrained over significant evolutionary distance, and thus it seemed reasonable that an analysis of the patterns of binding site constraint within bound regions might provide insight into their function.

We began with two measures of sequence constraint: (1) rates of pairwise substitution between D. melanogaster and its sister species D. simulans, and (2) PhastCons scores measuring constraint across 12 sequenced, closely and distantly related *Drosophila* species [57,58]. Because D. melanogaster and D. simulans are so closely related, there is essentially no ambiguity in alignments of their genomes. However, the small number of changes also limits our ability to detect differences in rates of evolution between classes of sequences by rates of pairwise substitution. PhastCons scores that employ a wider diversity of species, in contrast, have a much greater statistical power, but can be confounded by alignment error in those species that are more distantly related [59].

**Figure 15:**
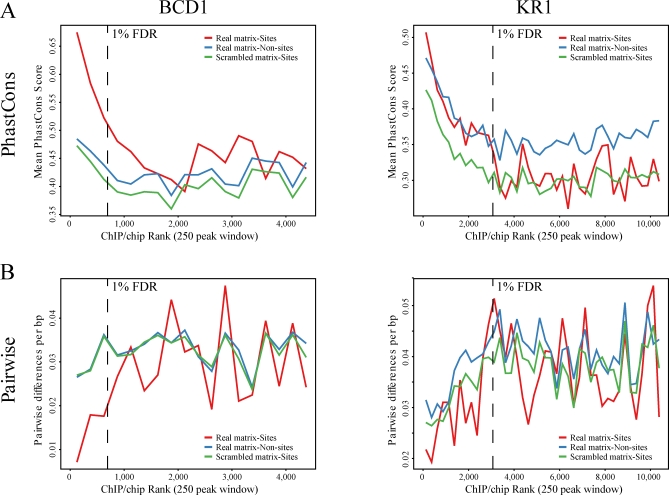
Recognition Sequence Conservation as a Function of Peak Intensity Conservation scores in predicted factor recognition sequences (*p*-value ≤ 0.001) (red lines), all remaining sequences (blue lines), and in sequences matching scrambled variants of the factors' recognition sequences (*p*-value ≤ 0.001) (green lines) in the 500-bp regions (±250 bp) around BCD1 and KR1 1% FDR peaks, in nonoverlapping windows of 250 peaks down the rank list to the 25% FDR cutoff. Panels in row (A) shows the mean PhastCons scores, and panels in row (B) the average pairwise differences per base pair between D. melanogaster and D. simulans. Gaps are ignored in the pairwise analysis. The 1% FDR cutoff is indicated by a vertical dotted line.

For each transcription factor, we examined constraint on recognition sequences in 500-bp primary peaks from 1% FDR regions, unbound regions, and short introns (see Table 3). We also examined both measures of constraint down the rank lists of bound regions for each factor (see Figures 15 and S12). In both analyses, we focused exclusively on recognition sequences in noncoding DNA (introns and intergenic sequences, with RNA coding genes excluded) so as to avoid the confounding effects of conservation of coding capacity.

**Table 3:**
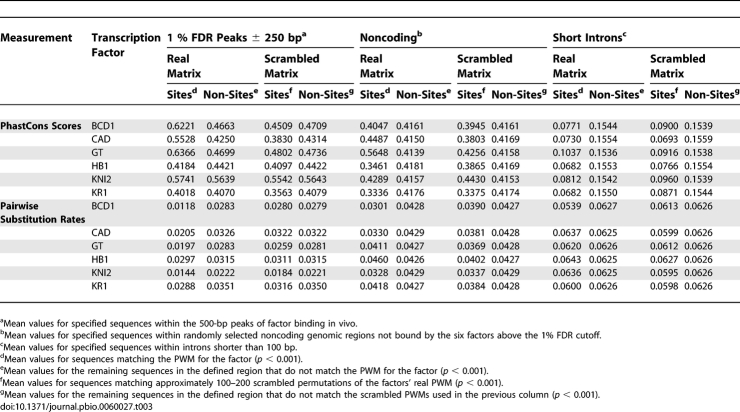
Conservation of Recognition Sequences

As shown in Table 3 and Figures 15 and S12, for each of the six factors, there is a general trend for recognition sequences in highly bound regions to be under the strongest constraint, followed by recognition sequences in poorly bound regions, recognition sequences in unbound regions, and recognition sequences in short introns. For example, for DNA sequences matching the KR PWM, their PhastCons scores are highest in the several hundred most highly bound regions (Figure 15A), and their mean PhastCons scores in 1% FDR regions are somewhat higher than those in unbound noncoding DNA and much higher than those in short introns (Table 3, PhastCons Scores). A similar trend, however, is observed for the remaining parts of the bound regions, outside of the specific factor recognition sequences, so the observed effect is at least in part due to overall higher levels of constraint in highly bound regions (“non-sites” data in Figures 15 and S12, Table 3).

To evaluate the extent to which these patterns of constraint were specific to recognition sequences for the factor, we therefore examined constraint on recognition sequences predicted after randomly permuting the order of columns of the specificity matrix for each factor.

For regions highly bound by BCD, CAD, and GT, none of the scrambled permutations produced recognition sequences that were as highly constrained as those from the real specificity matrix, and the average derived using many permutated PWMs was significantly lower than from the real PWM (Figure 15, Table 3). In fact, the average of the scrambled PWMs was similar to that of the remaining parts of the bound regions, outside of the specific factor recognition sequences (Figures 15 and S12, Table 3). Only in the case of BCD, however, did the patterns of constraint correlate with the in vivo DNA binding data because the additional constraint on recognition sequences, above the local background, dropped down the rank list, disappearing at around the 1% FDR threshold. For CAD and GT, the difference in conservation was consistent across the rank list all the way to the 25% FDR threshold, suggesting that the excess conservation may not be specific for bound recognition sequences.

For HB, KNI, and KR, there is even less evidence for specific conservation of recognition sequences because many of the permutations produced recognition sequences that were more conserved than the real sites, and the average score of these permutated PWMs were not significantly different from those of the real matrix at either highly or poorly bound regions (Table 3; Figure 15).

Overall, the comparative analysis adds further evidence that highly bound regions differ in character from poorly bound regions, but, with the exception of BCD, does not provide compelling evidence that the binding we observe contributes to organismal fitness.

### Discovery of Recognition Sequences for Additional Factors

Although the six factors studied here are the initiating regulators of A-P expression in the embryonic trunk, it is likely that other factors are involved in activating or otherwise regulating their targets. For example, several known targets of maternal and gap factors are also regulated by genes in the terminal system that controls expression in the head and tail [60–62].

To investigate whether other factors may be binding to the regions bound by the maternal and gap transcription factors, we systematically searched for sequences enriched in the regions surrounding the primary peaks for each of the six factors. As shown above, the recognition sequences of each factor are enriched in their respective bound regions, and these sequences are routinely recovered in de novo searches for enriched sequences in bound regions. However, for each of the six factors, the most strongly enriched sequence was the heptamer CAGGTAG/CTACCTG (Table S4). This “TAGteam” sequence has been previously reported to control the timing of preblastoderm transcription [63,64]. Although its precise role in activating transcription is only beginning to be understood [63,65], it is found in roughly 30% of bound regions and is concentrated in the most highly bound regions, emphasizing the broad role that it plays in early embryonic transcription. Furthermore, of all heptamers, CAGGTAG shows the greatest increase in interspecies conservation in bound regions relative to non-bound intergenic sequences.

## Discussion

The most striking feature of our in vivo binding data is that the maternal and gap transcription factors that regulate A-P patterning in D. melanogaster embryos bind to thousands of highly overlapping regions across the genome. We have rigorously established that the array hybridizations represent bona fide, sequence-specific binding of these transcription factors to DNA. For example, there is a high correlation between data derived using independent antibodies against the same factor; Q-PCR analysis validates the FDR estimates; and there is, on average, a strong enrichment of the recognition sequences for each factor in its bound regions. The extent of the binding is also consistent with earlier in vivo UV crosslinking experiments on a sample of known targets, unexpected targets, and transcriptionally inactive genes, which first predicted widespread, overlapping DNA binding by BCD and other early regulators [32,34].

### Determining the Functional Significance of Widespread Binding

Given that our ChIP/chip data identify several orders of magnitude more bound regions than the number of previously identified targets of these factors, the most immediate question is whether these bound regions are all functional. Several lines of evidence suggest that the bulk of the several hundred most highly bound regions are directly involved in regulating the transcription of neighboring genes. In particular, these most highly bound regions are preferentially found near genes transcribed in the blastoderm, these putative targets are enriched for patterned genes and genes with known roles in patterning and early development, and the most highly bound regions are preferentially conserved relative to other noncoding sequences (Figures 3–5, 7–8, 10–11, 15, S12, and Table 3). The highly bound regions also tend to be located within intergenic and intronic sequences (Figure 9), as expected for regulatory sequences.

All of these associations, however, dissipate down the rank lists for each factor, with an increasing percentage of more poorly bound regions mapping to genes that are not transcribed at this early stage of development and/or to protein coding regions or to noncoding regions that are less well conserved (Figures 3–5, 7–11, 15, S11, S12, and Table 3). This suggests that the poorly bound regions have different, or perhaps even no, function.

One possibility is that poorly bound regions regulate the transcription of adjacent genes, but more subtly than highly bound regions as it has been shown that many genes not directly involved in A-P patterning (e.g., housekeeping genes) show weak A-P patterns at stage 5 [35,66,67].

Another possibility is that the low levels of binding seen at stage 5 may presage stronger binding and transcriptional regulation of adjacent genes later in development. In support of this, binding of HB increases in the neuroectoderm of stage 9 embryos at a subset of regions bound at low levels at stage 5 (unpublished data), which, as genes become transcribed, likely results at least in part from a change in chromatin structure increasing access of factors to their recognition sequences [68–73].

A third possibility is that the observed binding is not involved in transcriptional regulation, but instead plays a role in regulating processes such as chromosome structure, DNA replication, or DNA repair. However, these six transcription factors have not been implicated in other cellular functions to date.

Finally, and in many ways most tantalizingly, some lower-level binding may be truly nonfunctional and simply result from transcription factors binding to randomly occurring target sequences that, precisely because they do not significantly affect gene expression, are not selected against. Indeed, it has long been proposed on thermodynamic grounds that transcription factors would bind at low, nonfunctional levels throughout the genome either via sequence-independent [74–76] or sequence-specific DNA binding [32]. However, even with these factors bound poorly to many thousands of regions across the genome, at any instant they could only bind to a small fraction of their recognition sequences within the genome, and they would still inevitably have an indirect function in the system by buffering the molecules available for binding within CRMs.

Determining which regions bound in vivo are functional and in what way(s) they function will be challenging. Our most reliable assay for sequences that regulate transcription—the construction of transgenic D. melanogaster embryos in which the sequence to be assayed is juxtaposed with a basal promoter and reporter gene—has several limitations. The assay only detects sequences that act independently of other sequences, whereas many bound regions are likely to augment the activity of other sequences or act redundantly [77,78]. Subtle or redundant regulatory activity is often difficult to detect in transgenes that use nonnative promoters and reporter genes. Finally, repressor, insulator, and other transcriptional regulatory activities require separate assays.

Comparative sequence analysis also has the potential to contribute to the dissection of the function of bound regions and the recognition sites within them. These analyses, however, can be extremely complex and occasionally misleading. It is common in published analyses of regulatory sequence conservation to assume that recognition sequences occur in a homogenous background of nonconserved sequences. But neither the assumption of neutrality nor that of homogeneity is appropriate. A substantial fraction of *Drosophila* noncoding DNA is under selective constraint—and presumably involved in some function [51] (Table 3). Thus simply observing that a collection of recognition sequences is conserved (i.e., evolves slower than the presumptive neutral rate), as has frequently been done in the literature, does not reliably establish that transcription factor binding to these sequences contributes to fitness. It is necessary instead to use methods that attempt to detect conservation of binding potential of particular recognition sequences [50,79]. Even this is complicated, however, by variation in rates of constraint that are correlated with genomic features that are in turn related to transcription. For example, noncoding sequences flanking genes transcribed in the embryo are more conserved than randomly selected noncoding sequences. Since highly bound sequences are also associated with genes transcribed in the embryo, it appears, often incorrectly, that recognition sequences in highly bound regions are preferentially conserved. We have used several methods to control for these effects, but they may still be susceptible to other confounding factors. Our analysis to date has only been able to establish for one of the six factors (BCD) that its recognition sequences are constrained above the background in the flanking DNA, even within the most highly bound regions (Figure 15, Table 3). For the other five factors, the results are ambiguous or not apparent (Figures 15 and S12, Table 3). This raises the unpleasant possibility that evolutionary constraint may not be as useful as generally believed for distinguishing functional targets of transcription factors.

### Targeting Transcription Factors to DNA

Our data suggest that the rules governing factor targeting in vivo are likely to be subtle and complex. Consistent with in vivo crosslinking analyses of other animal transcription factors [39,42,80–84], the more highly crosslinked regions in vivo do, on average, show greater enrichment of factor recognition sequences than poorly bound or unbound regions (Figures 12B, 12C, and S7), indicating that these factors' intrinsic DNA binding specificities play a role in determining the pattern of binding in vivo. Not only high-affinity recognition sequences, but also low-affinity sequences are enriched, suggesting that weaker sites help mediate binding (Figure 12B and S7).

As shown previously [32,34,42], however, in vitro DNA specificity alone cannot fully account for the distribution in vivo because many nonbound genomic regions contain higher densities of high-affinity recognition sequences than bound regions (Figures 12D and S7). Additional analyses indicate that factor targeting also depends on a local context established by other transcription factors: the degree of in vivo binding of a factor per number of specific recognition sequences at a given location is dependent on which other factors also bind the region in vivo (Figure 14). We do not know which factors are mechanistically responsible for establishing this context. The context could either be established by some of the A-P regulators themselves, or by one or more of the other factors whose recognition sequences are enriched in the bound regions (Table S4). In either case, the context establishing factors could act by cooperative interactions to increase A-P regulator DNA occupancy [85], or by increasing accessibility to factor recognition sequences locally via an effect on chromatin structure [70,86].

The idea that chromatin structure plays a dominant role is appealing as it provides a ready explanation for why we observe multiple factors being targeted to the same highly overlapping set of regions (Figures 6 and S4, Table 2). Each open chromatin region is expected to contain recognition sequences for many transcription factors because of the high frequency of such sequences throughout the genome. The factors would then be forced to bind the recognition sequences in open regions because regions elsewhere would not be available [70,86].

### Other Interpretations of In Vivo DNA Binding in Animals

Some of our conclusions agree with those of other recent studies of in vivo DNA binding by sequence-specific transcription factors in animals. For example, some of these other proteins are also observed to bind extensively to a large number of genomic regions [38,42,80,83,87,88]. But in some important regards, our analyses and conclusions differ.

Given that the earliest in vivo crosslinking studies of sequence-specific transcription factors established that, at least for some factors, genes are bound over a quantitative range that correlates with gene type, degree of gene regulation, and transcriptional state [34,78], it is very surprising that all recent analyses have ignored this quantitative information and instead classified genomic regions as either bound or not bound [38,39,42,80,82–84,87,88]. A range of experiments suggest that crosslinking and ChIP/chip signals broadly correlate with different levels of transcription factor occupancy [32,34] (Figures S1 and S2). The analyses presented in this paper clearly reinforce how useful it is to consider the relative level of transcription factor occupancy in studying the complex range of genomic regions bound by animal transcription factors in vivo.

Most analyses have either assumed that all regions bound in vivo must be functional targets or not actively considered whether a substantial fraction of bound regions may be nonfunctional [38,39,42,80,82–84,87,88]. Only a recent paper from the ENCODE Consortium [81] has seriously considered the possibility that a significant percent of in vivo binding may be nonfunctional, based on the lack of evolutionary constraint in bound sequences. However, the absence of constraint does not establish the absence of function, as it is well established that regulatory sequences can maintain their function in the absence of primary sequence conservation. We have shown that poorly bound regions lack many of the hallmarks of regulatory sequences.

Another recent study of in vivo binding by sequence-specific transcription factors in *Drosophila* measured DNA methylation patterns of transcription factor/DNA adenine methyltransferase (Dam) fusion proteins. Binding was assayed over a 2.9-Mb region of the genome in tissue culture cells for seven ectopically expressed factors, including BCD [89]. These fusion proteins were strongly targeted to a common set of “hot spots.” Because the factors have unrelated functions, it was proposed that hot spots are not classical *cis*-regulatory elements, but instead act either as sinks to sequester molecules, as mediators of interactions between distant genomic loci, or as unconventional enhancers at which many factors play only a minor role. The pattern of binding of endogenous BCD we observe in embryos, however, differs dramatically from that predicted by the methylation patterns in tissue culture cells. Within the 2.9-Mb region, only 16 1% FDR BCD bound regions are present. Of these, only five overlap the top 50 regions detected in the methylation assay, suggesting that the distributions mapped in tissue culture cells do not reflect binding by regulators normally expressed in other cells. In addition, we have mapped the binding of an additional 12 endogenous sequence-specific transcription factors in the early embryo that control D-V axis patterning and pair rule segmentation and which represent a broad range of transcription factor families (unpublished data). Together with the six maternal and gap A-P regulators studied in this paper, these endogenous factors do in fact frequently target the same short genomic regions, though they bind these regions at very different relative levels. These commonly bound regions, however, include most of the well-known CRMs active in the early embryo. We suspect that many animal CRMs are bound by a much larger number of factors than currently realized, though it remains to be determined what fraction of this binding is functional.

## Materials and Methods

### Antibodies.

Rabbit antisera were kindly provided by Sean Carroll (HB), Gary Struhl (BCD and CAD), Herbert Jackle, Ralf Pflanz, and Pilar Carrera (KR). Rabbit antisera for KNI was raised from a 6xHIS-tagged full-length KNI protein expressed in Escherichia coli. For each of the six transcription factors, two sets of antibodies that recognize nonoverlapping portions of the protein were affinity purified from rabbit antisera. The Gateway cloning and expression system (Invitrogen) was used to generate parts of each protein for affinity purification. Each affinity reagent consists of at least 100 amino acids that do not contain any significant similarity with any other protein in the D. melanogaster genome (no segment was used if it had greater than 20% identity to any other D. melanogaster protein, or perfect identity of ten amino acids or greater, as assessed by BLASTP [90]). To generate recombinant proteins, we cloned PCR-amplified fragments corresponding to the selected amino acid regions (below) using BP Clonase and the pDONR221 vector (Invitrogen). After sequence verification of the entire amplified product, the fragments were transferred to the 6xHis-tagged bacterial expression vector pDest17 using the LR-Clonase and subsequently verified by PCR. Anti-HB (HB1) and anti-HB (HB2) were purified against HB amino acids 1–305 and amino acids 306–758, respectively; anti-GT was purified using GT amino acids 182–353; anti-KR (KR1) and anti-KR (KR2) were purified against KR amino acids 1–230 and amino acids 351–502, respectively; anti-CAD was purified against CAD amino acids 1–240; and anti-KNI (KNI1) and anti-KNI (KNI2) were purified against KNI amino acids 130–280 and amino acids 281–425, respectively. The two affinity-purified sets of anti-BCD antibodies (BCD1 and BCD2) were purified against BCD amino acids 56–330 and 330–439, respectively, as described previously [34]. The monoclonal antibody H14, which recognizes RNA polymerase II CTD repeats phosphorylated at Ser 5, was obtained from CRP Inc. Further details on BDTNP antibodies and protein expression vectors are at http://bdtnp.lbl.gov/Fly-Net/.

### Crosslinking, chromatin isolation, and ChIP/chip.

The detailed protocol (Protocol S1) was used. Briefly, 2–3-h-old embryos (late stage 4 and early stage 5) were formaldehyde crosslinked, and chromatin was isolation by CsCl gradient ultracentrifugation as described previously [25,31,78,91]. The isolated chromatin was sonicated to an average size of about 600 bp prior to ChIP and dialyzed.

Protein A–Sephacryl 1000 beads were prepared based on a method described previously [92]. The larger pore size was found to give at least a 3-fold higher yield of crosslinked chromatin after immunoprecipitation. The chromatin solution was precleared by incubating with normal rabbit IgG and the protein A–Sephacryl 1000 beads. Factor IP reactions were carried out in duplicate by incubating 100 μg of chromatin with 0.5–3 μg of the appropriate antibody for 3 h or overnight at 4 °C; parallel control IgG IP reactions, also in duplicate, were carried out with normal rabbit IgG. The immunoprotein–chromatin complexes were captured by incubating with protein A–Sephacryl 1000 beads, followed by consecutive washes and then eluted with a buffer containing 1% SDS and 0.1 M NaHCO_3_ (pH 10.0). In each ChIP experiment, a portion of the chromatin solution corresponding to 1% of that used in the ChIP reaction was used as input DNA control. The DNA from this sample, along with the Factor IP and IgG control IP samples, was purified by phenol/chloroform extraction and ethanol precipitation after the protein/DNA crosslinks had been reversed by incubation at 65 °C.

Duplicate Factor IP, IgG control IP, and input DNA samples were amplified using a modified random primer-based DNA amplification protocol that gives significantly improved amplification consistency, particularly when the small quantities of genomic DNA recovered in our ChIP reactions (<0.5 ng) are amplified. After amplification, each DNA sample was fragmented with DNase I, biotinylated, and hybridized to Affymetrix *Drosophila* genomic tiling arrays [26]. Each array contains over 3 million oligo probes that cover the euchromatic portion of the genome at a resolution of about one per 36 bp on average.

### Primary array analysis.

ChIP/chip array data were processed using TiMAT, a Java- and R-based open-source software package developed by the BDTNP (http://bdtnp.lbl.gov/TiMAT/TiMAT2/). Array images were visually inspected for blemishes and artifactual bright spots, which were then masked to the array's median probe intensity value in the few cases necessary. Only oligonucleotides present exactly once in the D. melanogaster genome (release 4.0) were used for subsequence analysis. The complete set of six arrays from an experiment—Factor IP replicates, IgG control-IP replicates, and input DNA replicates (Figure 2A and 2B)—were scaled to a common median value and then quantile normalized against each other [93]. Replicates were averaged and log (base 2) ratio scores were calculated for Factor IP and IgG control IP arrays: log(mean Factor IP/mean input DNA) and log(mean IgG control IP/mean input DNA) (Figure 2C) [94]. These scores were then smoothed using a sliding window of trimmed means 675 bp in length (Figure 2D).

Next, FDR estimates were calculated by two methods. The first used an assumption of a symmetric window scores null distribution to compute *p*-values, then applied a multiple testing correction [95] to control the FDR. More specifically, the symmetric null method constructed a null distribution estimate by using window scores to the left of the mode of the full distribution and then reflecting these scores over the mode (Figure 2F). This approach is justified by the presumed relative scarcity of genomic regions enriched in the immunoprecipitation, and is a nonparametric variant on the approach of [30]. The second method simply computed the ratio of the number of windows scoring above a given cutoff in the IgG control IP data to the number in the Factor IP data—the assumption being that there are no true positives in the negative control data (Figure 2G).

Bound regions (called “intervals” in TiMAT) were then defined by first filtering out all windows with scores above the given FDR threshold, then collecting these into contiguous stretches of windows containing a minimum of ten windows, with a maximum allowable gap of 200 bp between any two adjacent windows (Figure 2E). Both 1% and 25% FDR thresholds were considered. The location of the maximum array hybridization within each bound region was determined and defined as its “peak window,” with the oligo having maximum intensity within each bound region defined as the “primary peak.” Local peaks of array intensity were identified within each bound region using a recursive algorithm that considers peak shape, height, and period. In this paper, for simplicity, only the largest (primary) peak in each bound region has been used in subsequent analyses, which in some cases removed secondary and tertiary peaks that show characteristics of CRMs.

Results, including oligonucleotide probe intensities, trimmed-mean window scores, bound region locations, peak magnitudes and locations, and nearby genes are reported in .sgr and .gff file formats as well as TiMAT's own text-based report files.

### Q-PCR validation of binding regions detected by ChIP/chip.

The bound regions analyzed by Q-PCR were selected arbitrarily throughout the symmetric null 1% FDR rank list for BCD and KR. KR bound regions between the symmetric null FDR score 1% and 25% thresholds were chosen with a bias towards the more poorly bound regions. The oligonucleotide primers and probes were designed to be as close as possible to the peak of each binding region, the majority falling within 200 bp of the peak. Q-PCR reactions were carried either using the random-prime–amplified input DNA and Factor IP DNA samples or the original Factor IP and IgG control IP samples, i.e., without random-prime amplification.

### BAC spike-in analysis.

Eight BAC plasmids, each containing about 170 kb of *Drosophila* genomic sequence were used. The BAC DNAs were mixed together at a relative molar concentration of one, four, ten, and 20, with two BACS at each concentration. The BAC DNA cocktail was then mixed with *Drosophila* whole-genomic DNA to generate two samples, one containing BACs at one, four, ten, and 20 times the molar concentration of genomic DNA, and the other at two, eight, 20, and 40 times. A total of 20 ng of each genomic DNA/BAC cocktail were random-prime amplified, and the resulting DNA samples were fragmented, biotinylated, and hybridized to chips following our protocol (above).

### Association of bound regions with target genes.

Bound regions were associated with the gene (from release 4.3 of the D. melanogaster genome) whose 5′ end was closest to the primary peak in the bound region. To identify the closest transcribed gene, the subset of release 4.3 annotations that completely overlap regions bound by RNA PolII in our ChIP/chip experiments was used.

### PWM construction.

For BCD, HB, GT, CAD, and KR, PWMs were constructed from unpublished SELEX data using MEME [96]. KNI was constructed from DNaseI footprint data contained in [40].

### Analysis of binding site enrichment.

Binding site positions were predicted using PATSER [97] with the indicated *p*-value cutoffs. Binding site enrichment was measured by dividing the density of sites in the bound regions by the density of sites in a control set of unbound sequences. The control set consisted of randomly selected noncoding sequences that did not overlap with 1% FDR regions for any factor.

### Evolutionary conservation of binding sites.

Evolutionary constraint on recognition sequences and bound regions was computed using 15 species (12 sequenced *Drosophila* species plus Anopheles gambiae, Apis mellifera, and Tribolium castaneum) PhastCons scores obtained from the Univeristy of California Santa Cruz Genome Browser (http://genome-test.cse.ucsc.edu/goldenPath/dm2/multiz15way/) and the pairwise D. melanogaster–D. simulans divergence was computed from LAGAN alignment of orthologous noncoding regions identified by a combination of BLAST and synteny. Mean constraints of recognition sequences in bound regions was compared to the same for recognition sequences in short (less than 100 bp) introns and in unbound noncoding regions. In addition, mean constraint was computed for recognition sequences predicted using randomly permuted PWMs for each factor in which the order of the matrix columns had been scrambled (permutations whose recognition sequences were enriched in bound regions were excluded to avoid permuted matrixes that were too similar to the unpermuted matrix). For the pairwise D. melanogaster–D. simulans comparisons, only substitutions, and not insertions or deletions, were considered.

## Supporting Information

Figure S1 Q-PCR Analysis Supports the FDR Estimates Regions of approximately 100 bp in size were Q-PCR amplified from crosslinked chromatin that had first been immunoprecipitated with anti-KR (A) antibodies or anti-BCD antibody (B) and then random-prime amplified. The immunoprecipitated DNAs analyzed were the same as those hybridized to the tiling arrays used to generate the ChIP/chip data in this paper. Q-PCR–amplified regions were selected from KR and BCD bound regions or from arbitrarily selected regions of the genome outside of the 25% FDR bound regions, which are expected to be either unbound or very poorly bound. Bound regions from the 1% FDR set were selected at random; KR bound regions between the 1% and 25% FDR threshold were selected based on being identified by both KR antibodies and intentionally biased to include regions scoring close to the 25% FDR threshold. The bound regions are ordered by rank of the peak window score, and the presumed unbound/poorly bound regions are shown to the right (*x*-axis). The enrichment of the amplicons in the immunoprecipitated DNA has been normalized by the enrichment of the same region from an equal amount of input DNA (*y*-axis). A fragment enriched by 1-fold (red line) is thus present at the equal concentrations in the treatment IP and input DNAs. Each vertical bar shows the mean of two independent immunoprecipitations, with the standard deviation indicated as well. All bound regions above the 1% FDR were enriched more than 1-fold and virtually all by more that 2-fold. For the KR bound regions between the 1% and 25% FDR threshold, 11 out of 16 showed enrichment greater than one (those enriched by less are indicated with an asterisk [*]). In contrast, only one region scoring below the 25% FDR was enriched by more than 1-fold (#), and that only in an immunoprecipitation by one of the two antibodies used. Thus the Q-PCR analysis is consistent with the FDR estimates.
(1.4 MB PDF)

Figure S2 ChIP/chip Array Intensity Correlates with Relative Enrichment in Immunoprecipitated DNA (A) ChIP/chip peak window scores of selected bound regions were compared to the regions enrichment in immunoprecipitated DNA as determined by Q-PCR. Q-PCR was conducted on samples either before (red points) or after (blue points) immunoprecipitated DNA had been random-prime amplified. The Q-PCR measured enrichment of random-prime–amplified DNA was calculated by normalizing against input DNA (see Figure S1). The enrichment of bound regions in unamplified immunoprecipitated DNA was calculated by normalizing against the enrichment of the same regions in IgG control immunoprecipitated DNA. The fold difference in enrichment between highly and poorly bound regions is larger in immunoprecipitated DNA before random-prime amplification than after. The fold difference as determined by array intensity is lower still, but cannot be directly compared to the Q-PCR data because it is averaged over a larger 675-bp window, which may reduce the enrichment measured. Nevertheless, the data indicate that there is a good correlation between enrichment detected by Q-PCR and the ChIP/chip array intensity scores for both the original immunoprecipitated samples (*r* = 0.94) and the random-prime–amplified sample (*r* = 0.71).
(B) Correlation between known concentrations of DNA and array window score. BAC DNAs from different genomic regions were combined at a molar ratio of one (light and dark green), four (light and dark blue), ten (light and dark yellow), and 20 (light and dark red), two BACs for each concentration (See Materials and Methods for details). The BAC DNA cocktail was then added to two separate genomic DNA samples, one aliquot of the cocktail being added such that the lowest concentration BAC was present at the same molar concentration as genomic DNA and the other aliquot at twice that concentration. These mixtures of genomic DNA and spiked-in BACs were random-prime amplified and hybridized to tiling arrays. The trimmed-mean window score for all windows spanning each BAC is plotted, as is the standard deviation of the trimmed-mean window scores. There is a good correlation between relative DNA concentration and mean window score (*r* = 0.84) despite likely errors in correctly determining the concentrations of BAC DNA. A 100% increase in the relative concentration of a BAC led to only an average only 31% increase in mean window score, again indicating that random-prime amplification and the array-based assay compress relative differences in DNA amount. The standard deviation of window scores are on average 16% of the mean for each BAC, indicating that the assay can correctly distinguish the majority of DNAs present at concentrations that differ by more than a factor of four.
(231 KB PDF)

Figure S3 Known *cis*-Regulatory Modules Tend to Be among the Regions More Highly Bound In Vivo The 1% FDR bound regions for each factor were each divided into cohorts based on primary peak window score (*x*-axis). For each cohort, the fraction of bound regions out of the total number of 1% FDR regions (red vertical bars) and fraction of bound regions in which the primary peak is contained within a CRM known to be regulated by the A-P factors (blue vertical bars) are shown. The number of bound regions in each cohort is given above the vertical bars.
(450 KB PDF)

Figure S4 Factors Bind with Quantitatively Different Specificities to Shared Target Regions Correlation of primary peak score between overlapping (within 500 bp) primary peaks for BCD1, CAD, GT, KNI2, and HB1. See Figure 6 for further details.
(3.9 MB PDF)

Figure S5 Highly Bound Genes Are Associated with Genes Transcribed and Patterned in the Early Embryo The percentage of 1% FDR primary peaks that are within 10 kb of the 5' end of a gene. Genes are divided into three categories: all genes (from genome release 4.3, March 2006), genes with known patterned expression (hand annotated based on Berkeley *Drosophila* Genome Project [BDGP] in situ images [35]), and transcribed genes (defined by our RNA Polymerase II ChIP/chip binding data, see Materials and Methods). Percentages are calculated in nonoverlapping windows of 100 peaks down the rank list to the 80% FDR threshold. The position of the 1% and 25% FDR cutoffs are indicated with vertical dotted lines. (A) shows the results for the CAD antibody, (B) for the GT antibody, (C) for the HB1 antibody, and (D) for the KNI2 antibody.
(1.9 MB PDF)

Figure S6 Genes That Control Development Are Highly Bound In Vivo The five most-enriched GO ([98]) terms in the 1% FDR bound regions for each factor were identified (enrichment measured by a hypergeometric test). The significance of the enrichment (−log(*p*-value)) of these five terms plus those for two negative controls (protein metabolism and mitosis) in nonoverlapping windows of 250 peaks are shown down to the rank list for CAD (A), GT (B), HB1 (C), and KNI2 (D) as far as the 80% FDR cutoff. The 1% and 25% FDR cutoffs are indicated by vertical dotted lines.
(534 KB PDF)

Figure S7 Recognition Sequences Are Modestly Enriched in Bound Regions (A) Sequence logo representing the PWMs derived from SELEX data (made with seqlogo [99]).
(B) Fold enrichment of matches to the PWM from (A) in nonoverlapping windows of 100 bp across the 1% FDR primary peaks, with the peaks located at position zero on the *x*-axis. PWM matches shown are divided in subsets based on the *p*-value of their match to the matrix.
(C) Fold enrichment of matches to the PWM from (A) in the 500-bp regions around (±250 bp) primary peaks in nonoverlapping windows of 250 peaks down to the 25% FDR cutoff. The 1% FDR cutoff is indicated as a vertical dotted line. As in (B), matches are divided based on the significance of their match to the matrix.
(D) shows the distribution of the number of sites in the 500-bp regions (±250bp) around 1% FDR primary peaks and, for comparison, randomly selected noncoding genomic sequence. A match to the matrix in this panel is defined here as a *p*-value of ≤0.001.
(1.1 MB PDF)

Figure S8 GC Bias Not Due to Hybridization For all windows in BAC regions, the mean window score is shown against the window GC content as vertical bars (error bars show the standard error of the mean). The number of windows in each GC bin is shown as a blue line.
(275 KB PDF)

Figure S9 Enrichment of Randomly Permuted PWMs in Bound Regions Enrichment of matches to a randomly permuted version of the PWM (*p* ≤ 0.001), in 100-bp nonoverlapping windows across 10 kb (±5 kb) around 1% FDR primary peaks, for each factor. Enrichment calculated as described in Materials and Methods.
(520 KB PDF)

Figure S10 Enrichment of Recognition Sequences in Regions Bound by Other Factors Enrichment of matches to each PWM (*p* ≥ 0.001) in 500 bp (±250 bp) around 1% FDR primary peaks for each factor, after removing any peaks within 250 bp of a 25% FDR primary peak for the factor relating to each PWM, with the exception of enrichment for a PWM for a factor in bound regions for the same factor. Enrichment is displayed as a –log(*p*-value) from the binomial test as a grey scale, with white being the most enriched.
(270 KB PDF)

Figure S11 Enrichment of Recognition Sequences in Protein Coding Regions Enrichment of matches to a PWM (*p* ≤ 0.001) for each factor in 250-bp nonoverlapping windows across 10 kb (±5 kb) around 1% FDR primary peaks. Four collections of peaks are shown: those in all 1% FDR bound regions, those in coding regions, those in a noncoding sequence, and those in the most poorly bound quartile of noncoding regions.
(507 KB PDF)

Figure S12 Recognition Sequence Conservation as a Function of Peak Intensity Conservation scores in predicted factor recognition sequences (*p*-value ≤ 0.001) (red lines), all remaining sequences (blue lines), and in sequences matching scrambled variants of the factors' recognition sequences (*p*-value ≤ 0.001) (green lines) in the 500-bp regions (±250 bp) around CAD, GT, HB1, and KNI2 1% FDR peaks, in nonoverlapping windows of 250 peaks down the rank list to the 25% FDR cutoff. Panels in rows (A) and (C) show the mean PhastCons scores, and in rows (B) and (D), the average pairwise differences per base pair between D. melanogaster and D. simulans. Gaps are ignored in the pairwise analysis. The 1% FDR cutoff is indicated by a vertical dotted line.
(775 KB PDF)

Protocol S1 ChIP/chip Protocol (195 KB PDF)

Table S1 The 1% FDR Bound Regions (2.8 MB XLS)

Table S2 The 25% FDR Bound Regions. (12.0 MB XLS)

Table S3 Number of Bound Regions within 10 kb of the 5′ End of Genes That Gives Rise to miRNAs (37 KB XLS)

Table S4 De Novo Motifs Identified in Bound Regions (48 KB XLS)

## Abbreviations

A-P: anterior–posterior
BAC: bacterial artificial chromosome
(BCD: Bicoid
BDTNP: Berkeley Drosophila Transcription Network Project
CAD: Caudal
ChIP/chip: chromatin immunoprecipitation coupled with DNA microarray hybridization
CRM: *cis*-regulatory module
D-V: dorsal–ventral
FDR: false-discovery rate
GO: gene ontology
GT: Giant
HB: hunchback
IgG: immunoglobulin G
IP: immunoprecipitate
KNI: Knirps
KR: Krüppel
miRNA: microRNA
PWM: position weight matrix
Q-PCR: quantitative polymerase chain reaction
SELEX: in vitro selection
