# Correction: Transcription Factors Bind Thousands of Active and Inactive Regions in the Drosophila Blastoderm

**DOI:** 10.1371/journal.pbio.0060190

**Published:** 2008-07-29

**Authors:** Xiao-yong Li, Stewart MacArthur, Richard Bourgon, David Nix, Daniel A Pollard, Venky N Iyer, Aaron Hechmer, Lisa Simirenko, Mark Stapleton, Cris L Luengo Hendriks, Hou Cheng Chu, Nobuo Ogawa, William Inwood, Victor Sementchenko, Amy Beaton, Richard Weiszmann, Susan E Celniker, David W Knowles, Tom Gingeras, Terence P Speed, Michael B Eisen, Mark D Biggin

Correction for:

Li Xy, MacArthur S, Bourgon R, Nix D, Pollard DA, et al. (2008) Transcription factors bind thousands of active and inactive regions in the Drosophila blastoderm. PLoS Biol 6(2): e27. doi:10.1371/journal.pbio.0060027

The information in Table 1 for RNA polymerase II was incorrectly given for the form of the enzyme unphosphorylated at the C-terminal tail, which is recognized by the 8WG16 monoclonal antibody. The corrected version of the Table below gives the intended information for the enzyme phosphorylated at the C terminus, which is recognized by the H14 monoclonal antibody.

**Table 1:**
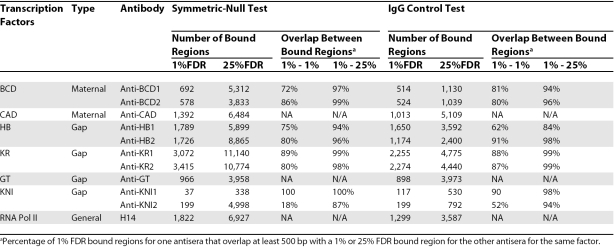
Number of Regions Bound by Transcription Factors

