# Supplementary material for: Transcription Factors Bind Thousands of Active and Inactive Regions in the Drosophila Blastoderm
Source: PLoS Biol. 2008 Feb 12;6(2):e27. doi: 10.1371/journal.pbio.0060027 (PMC2235902; doi:10.1371/journal.pbio.0060027)
Supplement: Supplementary file 1 [file pbio.0060027.sd001.pdf]

ChIP-chip Protocols

November, 2007 version

Berkeley Drosophila Transcription Network Project
Lawrence Berkeley National Laboratory

I. Formaldehyde Crosslinking *Drosophila* Embryos and Chromatin Purification.

Based on Toth, J. and Biggin, M.D. (2000) Nucleic Acids Res., 28. e4.

i. Formaldehyde Crosslinking of *Drosophila* Embryos

1. Prepare crosslinking solution by combining hexane with 10X PBS and 37% formaldehyde so that the final concentrations are 5% formaldehyde and 1X PBS. Shake vigorously and then mix with a stir bar for at least 30 minutes before use. Be sure to use only the top layer for crosslinking embryos.

10X PBS	/1L
1.37 M NaCl	80g NaCl
27 mM KCl	2g KCl
43 mM Na_2HPO_4	6.10g Na_2HPO_4
14 mM KH_2PO_4	2g KH_2PO_4

adjust pH to 7.3 and autoclave

<u>5% Formaldehyde saturated hexanes</u>
210.5 ml hexanes
37 ml 37% formaldehyde
27.5 ml 10X PBS
Prepare this mixture on the day of crosslinking. Aliquot the top (hexanes) layer to a separate container if small amounts are needed during the course of several hours.
* modification of Beta-galactosidase staining protocol from Christian Klambt

2. Collect properly staged embryos on double nitex mesh. Wash away yeast and dechorionate embryos for 2 minutes in 50 ml of 50% bleach per 5 g of embryos. Pour into small mesh and wash extensively.
3. Transfer embryos to a cup with mesh on the bottom. Completely immerse the embryos in a beaker of isopropanol and shake back and forth to disperse clumps. Remove cup and dry embryos as much as possible with paper towels beneath the mesh.
4. Transfer embryos to a 50mL Falcon tube containing 10 ml formaldehyde saturated hexanes per gram of embryos. Shake vigorously for 5 minutes at room temperature. Allow the embryos to settle and pour off as much of the hexanes as possible.
5. Resuspend embryos in the same volume of 1X PBS, 0.5% Triton X-100 and shake vigorously at room temp for 5 minutes. Pour onto nylon mesh and repeat with fresh buffer. Embryos should clump at first but then eventually become monodispersed. Dry embryos as much as possible and transfer them to plastic tubes; the amount of embryos for each tube should be enough for a $CsCl_2$ gradient in the chromatin purification step and depend on the stage of the embryos collected. Flash freeze embryos in liquid nitrogen.

ii Formaldehyde Crosslinked Chromatin Purification

Use autoclaved glassware and stir bars. Set up the required amounts of $CsCl_2$ gradient solutions so that they will be ready when needed

1. Thaw the frozen embryos. To each embryo sample, which should contain enough embryos for one $CsCl_2$ gradient (check table at end of the protocol for amount of embryos according to their stages), add 35 mls of cold NIB buffer + DTT + PMSF (shake hard to break the clumps, and to decrease embryos sticking to tube wall).
2. Transfer embryos to a glass dounce tube, and dounce the embryos at 8,000 rpm for one stroke and two strokes at 7,000 rpm using a motor – drive homogenizer system (Glas-Col).
3. Transfer to a large hand held dounce and dounce five times using A size pestle.
4. Pour into an SS34 tube and add 0.5 ml 20% Triton X100 to a final concentration of 0.3% and put on rotating wheel for 10 minutes. Spin for 15 min at 4,000 rpm at 4°C.
5. Pour off supernatant and add 5ml Nuclear Lysis Buffer (+ 1 mM PMSF) and loosen pellets by swirling gently.
6. Transfer to a small (B pestle) dounce and completely homogenize with several strokes.
7. Pour into a 15 ml falcon tube, sonicate for 20 seconds at setting 1.5 in a Branson sonifier 450 (settings may vary depending on type of sonicator) to partially fragment chromatin. This mild sonication step is not to reduce the chromatin fragment to sizes desired for ChIP, but rather to decrease viscosity of the sample in the following step with the addition of detergent.
8. Transfer to a clean SS-34 tube. Add 1.8 ml 20% SDS (to a final conc. of 3%) and quickly vortex.
9. Add 1.2 ml 20% Sarkosyl, and 1.2 ml 20% Triton X-100. Incubate on rotator at room temperature for 10 minutes.
10. Spin at 4,000 for 10 minutes in an SS-34 rotor. There should be a small pellet of vitelline membranes and unlysed cells.
11. Set up $CsCl_2$ gradients (check table at end of protocol for volumes of $CsCl_2$ solutions to each gradient), and layer the lysed nuclei on top of them.
12. Stop the ultra spin after the appropriate length of time. Secure each SW28 tube in a clamp and locate the gelatinous grey band (which is the chromatin) about 3 cm from the bottom of the tube. Gently insert an 18 1/2 gauge needle attached to a 5 ml syringe 0.5 – 1 cm below this band. Slowly pull the plunger and remove this material, collect 2-3 ml for each gradient.
13. Transfer the material to Spec 2 dialysis bags and dialyze against 0.1 % Sarkosyl, 2mM EDTA, 20 mM Tris pH 7.9 + 1 mM PMSF at 4°C for 2 hrs, repeat 2 more times with fresh buffer. The chromatin appears as aggregates at this stage, but will be solublized after sonication.
14. Adjust the Sarkosyl concentration in the chromatin solution to 0.5%. Sonicate the chromatin to average of ~750 bp. Power and duration of sonication depends on the sonicator, and have to be determined empirically.
15. Store chromatin at -80°C

Buffers and solutions for chromatin purification

NIB buffer	1L	
0.3 M Sucrose ultrapure		205.4g
15 mM NaCl		0.88g
5 mM $MgCl_2$		1.02g
15 mM Tris pH 7.5		7.5 ml of 2M Tris pH 7.5
60 mM KCl		4.48g
0.1 mM EDTA		0.2 ml of 0.5M EDTA
0.1 mM EGTA		.038g

Filter sterilize using a 0.2 µm filter and store at 4˚C. Add DTT to 0.5 mM and PMSF to 1 mM just before use.

Nuclei Lysis Buffer	1L	
100 mM NaCl		20ml of 5M
10 mM Tris pH 7.9		5 ml of 2M
1 mM EDTA		2 ml of 0.5M
0.1% NP-40		1 ml of pure NP-40

Filter sterilize using a 0.2 µm filter and store at 4˚C. Add PMSF to 1 mM before use.

$CsCl_2$ gradient buffer	1L	
2% Sarkosyl		20 g
1 mM EDTA		0.372 g

Parameters for chromatin purification:

Embryos (grams):		
4-5 hour	1-3 g	5-8 g
5.5-7.5 hour		2.5-5 g
8-10 hour	0.3-1 g	1.5-2.5 g
0-12 hour		5 g
Embryo homogenization		
Nuclei Irradiation buffer	13 ml	35 ml
Nuclear Lysis		
Nuclear Lysis buffer	1.7 ml	5 ml
20 % SDS	0.6 ml	1.8 ml
20 % Sarkosyl	0.4 ml	1.2 ml
20 % Triton X 100	0.4 ml	1.2 ml
total volume	*3.9 ml*	*11.8 ml*
Ultracentrifugation:		
Beckman rotor	SW41	SW28
Run speed/time (20˚C)	37K/~24hr	25K/~40hr
$CsCl_2$ steps:		
1.5 g/ml	2.6 ml	8.5 ml
1.4 g/ml	2.6 ml	8.5 ml
1.3 g/ml	2.6 ml	8.5 ml

$CsCl_2$ preparation (to make 400 ml of each solution)

Density	gram CsCl	mls Buffer*
1.50 g/ml	266.8	333.2
1.40 g/ml	213.5	346.5
1.30 g/ml	160	360

* Buffer should contain final concentrations of 2% Sarkosyl, 1mM EDTA

II. Chromatin Immunoprecipitation Using Chromatin from Formaldehyde Crosslinked *Drosophila* Embryos.

Based on Toth, J. and Biggin, M.D. (2000) Nucleic Acids Res., 28. e4.

Chromatin IP (ChIP) is carried out using chromatin that has been isolated from formaldehyde crosslinked embryos and sonicated to an average fragment size of 750 bp. Use 100 µg of chromatin for each ChIP reaction.

1. Just before use, spin the chromatin solution (in TE + 0.5% sarkosyl) in a micro centrifuge at full speed for 15 min at 4°C. Transfer the chromatin solution to new Eppendorf tubes.
2. Add 5x IP buffer to the chromatin solution at 1 : 4 ratio, to final concentration of 1x.
3. Add 7 µl of normal rabbit IgG (0.4 µg/µl, Santa Cruz BioTechnology)/100 µg chromatin. Incubate 30 min on ice.
4. Transfer the sample to an Eppendorf tube containing protein-A sephacryl-1000 beads, use 15 µl (packed volume) of beads for each 100 µg of chromatin. Incubate 1 hr on a rotator.

   <u>Preparation of protein-A Sephacryl beads:</u> beads for the above step are prepared as follows: wash beads twice with 1x IP buffer, and after finishing washing, remove buffer and leave just the beads in the tube. see Appendix for preparation of protein-A Sephacryl beads.
5. Spin in a micro centrifuge at full speed for 15 mins., transfer the precleared chromatin solution to a 15 ml falcon tube.
6. Dilute chromatin to 100 µg/1.2 ml with 1x IP buffer, add 4 µl 50 mg/ml BSA for each one ml of diluted chromatin, and add PMSF to 1 mM. Save 24 µl of the chromatin solution as **Input** sample (representing 2% of total input DNA).
7. For each IP or negative control IP sample, use 1200 µl of the diluted chromatin (~100 µg). For IP samples, add 0.5 – 3 µg of purified polyclonal antibody, or appropriate amount of monoclonal antibody or antiserum.

   For control IPs, add an equivalent amount of normal IgG, or preimmune serum. Incubate the samples on ice for 3 hr, or overnight at 4°C.
8. Spin the samples in a micro centrifuge at full speed for 15 mins. at 4 °C, Transfer supernatant to tubes containing 100 µl of the protein A-Sephacryl suspension.
   Incubate 30 mins. on a rotator.

   <u>Prepare Protein A beads</u>
   wash beads 2x with 1 ml 1xIP buffer + 200 µg/ml BSA. Resuspend beads in 1xIP buffer with BSA at a volume of 100 µl for each 10 µl beads.
   Use 100 µl of the protein A beads suspension for each IP reaction.
9. Pellet the beads by spinning the samples at 4K rpm for 1 min. in a micro centrifuge, discard supernatant.
10. Resuspend beads in 1. 4 ml 1x IP buffer, transfer to a new tube, rotate at room temp for 5 mins., pellet beads, discard supernatant – this is the 1st wash.
11. Carry out the following consecutive washes at room temperature similar to step 10, except do not change the tube:

    1x in IP buffer, 10 min
    2x in 0.5 M IP buffer, 10 min each*
    1x in LiCl buffer, 10 min*
    1x with TE, 10 min

    *Note: if the antibody binding reaction is incubated overnight, increase these washing steps to 15

- 20 min.

12. Resuspending the pellet in 1 ml TE, transfer to a new tube, and spin down the beads. Discard supernatant. Remove the remaining buffer in the beads with a 30G1/2 needle attached to a 1 ml syringe.
13. Add 150 µl elution buffer, put on a rotator for continuous mixing for 15 min at room temperature, spin at 4K rpm for 1 min, transfer supernatant to a new tube.

    Repeat the elution step, and combine the supernatant.
14. To each sample, add 30 µl of the following PK solution:

    15 µl 1 M Tris pH 7.5
    7 µl 0.5 M EDTA
    4 µl 5 M NaCl
    4 µl 10 mg/ml Proteinase K (Invitrogen)

    <u>For input sample (saved from step 6)</u>, add 235 µl H_2O, 15 µl 20% SDS, and 30 µl of the above PK solution
15. Incubate all the samples at 55°C, overnight
16. Transfer the samples to 65°C, incubate for 6 hrs to reverse crosslinks
17. DNA purification.

    add 30 µl 3M NaOAc, and 20 µg glycogen to each sample.
    Extract once with phenol:chloroform
    Extract once with chloroform.
    Ethanol precipitate at -80°C for 4 hrs or more.
    Spin 15 mins.
    Wash with 75% ethanol twice. Remove as much ethanol as possible after the last wash.
    Dry the DNA pellet at room temperature ~10 min.
18. Resuspend in TE (10mM Tris, 0.1 mM EDTA): 20 µl for IP samples, and 40 µl for Input DNA samples (2% of total DNA). The samples are now ready to be analyzed by PCR, or processed for chip analysis.

<u>Buffers and solutions:</u>

<u>1x IP buffer</u>
10 mM TrisCl pH 8.0
1 mM EDTA
150 mM NaCl
0.5% Triton-X100
0.1% Nadeoxycholate
0.5% Sarkosyl
add BSA and PMSF as indicated in procedure

<u>5x IP buffer</u>
50 mM TrisCl pH 8.0
5 mM EDTA
750 mM NaCl
2.5% Triton-X100
0.5% Nadeoxycholate
0.5% Sarkosyl

LiCl buffer
10 mM TrisCl pH 8
1mM EDTA
250 mM LiCl
1% NP40
1% Nadeoxylcholate

TE
10 mM Tris-Cl, pH8.0
0.1 mM EDTA

Elution buffer
0.1 M $NaHCO_3$ (pH 10)
1% SDS

Elution buffer should be made fresh each time from $NaHCO_3$ (2x) stock solution, which is stored in aliquot at –20°C. [a solution of 120 mM $NaHCO_3$+80 mM $NaCO_3$ has a pH around 10)

Other needed reagents
Chromatin, purified through $CsCl_2$ gradient, sonicated to average length of less than 1 kb.
Antibodies, affinity purified
Normal IgG as controls
Protein A –agarose beads*
BSA
Glycogen

*We use protein A –sephacryl 1000 beads prepared in house. This seems to work better than commercial protein A-agarose beads for critical applications.

III. DNA Amplification and Hybridization to Genomic DNA Tiling Array

i. DNA Amplification

This protocol is derived from an Affymetrix Protocol with the following modifications: i) A modification to the original primer sequence is introduced to avoid primer dimer formation, ii) 15x higher primer A concentration accompanied with different purification procedures following random priming, iii) higher sequenase concentration, iv) more random priming cycles (4 vs. 2), v) some changes in PCR conditions, and some other adjustments. These modifications together improve the DNA amplification efficiency and consistency. With this protocol, highly reproducible amplification can be obtained even when as little as 0.5 ng Drosophila whole genomic DNA is used.

For a typical ChIP reaction that uses ~100 µg chromatin (corresponds to ~1x10^8 cells), resuspend the DNA samples after the final ethanol precipitation step in small volumes (10-20 µl). Normally, use at least 50% of the IPed material for amplification. But if the efficiency of ChIP is high (e.g. if more than 0.1% of target sequenced is IPed), a smaller portion of the IPed sample can be used. The amount of input DNA used can vary from 5-200ng without affecting the results. But, typically, it can be determined based on the ChIP efficiency of a known target sequence, e.g., if 0.2% of the target sequence is IPed based on a PCR analysis, use an mount corresponding to 0.2% (200 ng) of input DNA for each amplification reaction.

1. Set up first round reaction as follows:

__µl	DNA
__µl	H2O
4 µl	5x sequenase buffer
4 µl	200 µM primer A
18.5 µl total	

   primer A (new version): GTTTCCCAGTCACGGTC(N)9

2. Carry out the reactions using the following cycle conditions (total 4 random priming cycles) using a Perkin Elmer 9600 or 9700 PCR machine.

   i) 95°C 4 min
   snap freeze sample (i.e. transfer sample to ice)

   ii) 10°C 5 min, or longer if more time needed to finish adding NTP mix or sequenase
   once cycler temperature reaches 10°C, place snap frozen samples back in cycler, and add 1.6 µl NTP mix (only the first of the four random priming cycles) and 1 µl of 6x diluted sequenase (USB Corp).

   iii) ramp to 37°C over 9 -10 min (1°C/20sec) [With a PE 9600 PCR machine, the ramp time can be set directly to 9 min; with the 9700 PCR machine, set the ramping speed at 3%, for 60 µl reaction (even though the actual volume is less), it will take 10 min to reach 37°C]

   iv) 37°C hold for 8 min

   Repeat (i-iv) 3 more times.

   NTP mix (per reaction):

0.1µl	20 mg/ml BSA
1 µl	0.1 M DTT
0.5 µl	25 mM dNTPs

   6x diluted sequenase
   dilute sequenase with sequenase dilution buffer

3. Purification of DNA samples from the 1st round: add 20 µl TE to each sample, pass the samples through two Amersham microspin S-300 HR columns sequentially by following the manufacturer's instructions (e.g. first remove buffer from column by spinning column at 3 krpm for 1 min in micro centrifuge; then load sample; and spin at 3 krpm for 2 min; save flow through). For the second column, after removing buffer in the column, re-equilibrate the column by adding 300 µl of 10 mM Tris/HCl, pH 8.5, followed by a 1 min centrifugation, and then load the sample. The purified DNA is ready for PCR amplification.

4. 2nd round PCR amplification: (It is worth checking the amplification first with 7 µl of sample from 1st round in 20 µl reaction, if not enough amplification obtained, use more cycles).

   35 µl of the DNA sample from the 1st round
   10 µl 10X PCR buffer
   3 µl 25 mM $MgCl_2$
   1.5 µl 25 mM dNTPs
   4 µl 100 µM Primer B (new version: GTTTCCCAGTCACGGTC)
   44.5 µl H_2O
   <u>2 µl 5U/µl Taq (Stratagene Taq2000)</u>
   Total reaction volume 100 µl

   Cycle conditions (total 30 – 35 cycles):

   15 cycles of
      95°C 30 sec
      45°C 30 sec
      55°C 30 sec
      72°C 1 min

   15 -20 cycles of
      95°C 30 sec
      45°C 30 sec
      55°C 30 sec
      72°C 1 min + D5"
   Hold 4°C

5. Purify amplified DNA. Use Qiagen PCR purification kit. Elute DNA using 35 µl H_2O. Check DNA concentration by measuring OD 260nm. Up to 12 µg of DNA can be obtained for each sample.

ii. DNA Fragmentation

Since the efficiency of DNase I digestion is sensitive to a number of conditions (for example purity of DNA, the vendor and lot of DNase I), the exact amount of DNase I to be used needs to be determined empirically.

1. Dilute DNase I (1U/µl; Epicentre) 15x into 1X One-Phor-All buffer (Roche)
2. Set up reactions as follows:

2.5-3 µg	Amplified DNA
? µl	H_2O
3.28 µl	10x One Phor-All buffer
<u>2.2 µl</u>	15X diluted DNase I
35 µl total	

3. Incubate for 5 min at 37°C

4. Inactivate DNase I at 99°C for 10 min, then place on ice.
5. Check 10% rxn on 1.5 % agarose gel, the bulk of DNA should be at 50-100 bp. If not, repeat the digest.

iii. TdT labeling

1. Set up reaction:

31.2 µl	DNase I treated DNA
10 µl	5x TdT buffer
5 µl	25 mM $CoCl_2$
3.6 µl	1 mM biotin-ddATP
0.18 µl	TdTase (Roche)
50 µl total	

2. Incubate at 37°C for 2 hr

iv. Hybridization to chips

1. Dilute 2x MES-triton to 1x, inject 200 µl to each chip
2. Prehybe ~ 1 hr in the Affymetrix hybridization oven at 45 rpm and 45°C
3. Mix 200 µl hybridization cocktail with 50 µl of the sample from TdT reaction.
4. Boil sample for 10 min, transfer to a 45°C temperature block, incubate for 10 min
5. Spin in a microcentrifuge at maximum speed for 3 min
6. Inject 200 µl of the sample in to the chip.
7. Carry out hybridization in the hybridization oven at 45 rpm and 45°C for 18 hrs
8. At end of hybridization, save the hybridization solution and store at -80°C so that rehybridization can be carried out if necessary; the chips are ready to be processed.

Hyb. cocktail	each chip	final
20.83 µl	12x MES	1x
150 µl	5 M TMAC	3 M
3 µl	3 nM B2	30-50 pM
2 µl	herring sperm DNA (10mg/ml)	100 µg/ml
5 µl	1% Triton	0.02%
18.67 µl	H_2O	
200 µl total		

<u>12X MES</u> 1 liter

70.4 g MES free acid monohydrate
193.3 g MES-Na
800 ml water, mix and adjust to 1000 ml
pH should be between 6.5 and 6.7, do not adjust
filter as above

2x MES-triton	10 ml
1.66 ml	12x MES
3.44 ml	5 M NaCl
0.8 ml	0.5 M EDTA
0.2 ml	1% triton-x100
3.9 ml	H_2O
	10 ml

Filer sterilize, store at 4°C, shield from light

V. Washing / staining

Use an Affymetrix GeneChIP Fluidics Station to process the hybridized chips following the wash/stain -protocol EukGeWS2v4.

Materials and Solutions

10mg/ml n.goat IgG**:** resuspend 10 mg in 1 ml 150 mM NaCl, aliquot, store at 4°C, and long term at -20°C

0.5 mg/ml biotinylated anti-streptavidin: 0.5 mg resuspend in 1 ml H_2O, aliquot, store at 4°C, and long term at -20°C

Wash buffer A	(1 liter)
300 ml	20x SSPE
1 ml	10% Tween 20
700 ml	H_2O

filter sterilize

Wash buffer B	(500 ml)
41.6 ml	12xMES
2.6 ml	5M NaCl
0.5 ml	10% Tween20
455.2 ml	H_2O

filter sterilize and store at 4°C, shield from light

2x stain buffer	(50 ml)
8.35ml	12x MES
18.5ml	5 M NaCl
0.5ml	10% Tween20
22.65ml	H_2O

filter sterilize and store at 4°C

SAPE sol	(1200 µl each chip)
600µl	2x MES stain buffer
48 µl	50 mg/ml BSA
12 µl	1mg/ml SAPE
540 µl	dd H_2O

Ab solution	(600 µl each chip)
300 µl	2x MES stain buffer
24 µl	50 mg/ml BSA
6 µl	10 mg/ml n. Goat IgG
3.6 µl	0.5 mg/ml biotinylated antibody
266.4 µl	H_2O

For each array, need 1 tube of 600 µl of Ab solution and two tubes each containing 600 µl SAPE

vi. Scanning Chips

Scan chips using Affymetrix GeneChip scanner

APPENDIX

Coupling of Protein A to Sephacryl S1000 Resin

Reference: Hornsey VS, Prowse CV, Pepper DS. Reductive amination for solid-phase coupling of protein. A practical alternative to cyanogen bromide. Journal of Immunological Methods, 93 (1986): 83-88.

rProtein A is from Pierce. Reconstitute by resuspending 5 mg in 1 ml H2O. (even though it is said that after reconstitution protein A can be stored at 4°C for up to 1 month, we found some degradation already occurred (10%?) following storage through weekend).

Protein A / beads ratio: 1.5 mg protein/ml beads.

Activation

1. Wash Sephacryl S-1000 (Amersham Pharmacia) 3-5 times with 0.1 M NaOAC pH4.7. Use 50 ml Falcon tube, and resuspend beads at 5 ml buffer/ml beads. Pellet the beads by spinning at 0.5 krpm for 30 seconds in a Beckman J6 centrifuge.

2. Resuspend beads in 3mM sodium periodate in 0.1 M NaOAC pH4.7 (5 ml/ml beads), mix end-over-end for 15 min at room temperature IN THE DARK.

3. Wash with H_2O (10ml/ml beads), and 3 times with 0.1 M trisodium citrate in 50 mM Sodium Carbonate pH10 (10 ml/ml beads).

   Oxidized gel can be stored in distilled water at 4^oC up to 4 mo. (NaN_3 may be added).

Protein Coupling

4. Dilute Protein A into fresh pH10 buffer that contains 5mM ascorbic acid (For each ml beads, use 0.3 ml 5 mg/ml protein A, and 5 ml pH10 buffer + 0.25 ml 100 mM ascorbic acid). Add this solution is to the settled gel, and incubate for 20 hrs at room temperature in dark with mixing.

5. Let the gel to settle. Remove supernatant and resuspend beads in pH10 buffer 5 ml/ml beads), and transfer to a empty BioRad chromatography column. This and all wash steps below are carried out in the column.

Blocking

6. Gel is resuspended with 1M Ethanolamine pH9 containing 5 mM ascorbic acid (5 ml/ml beads). Incubate with mixing for 1 hour at room temperature.

7. Gel washed successively with 5 ml /ml beads of each:

   a. Distilled water

   b. 0.1M glycine, pH3.0; incubate 30 min while mixing.

   c. Distilled water

d. 1M Ethanolamine pH9

e. 5M Urea

f. 3M Potassium thiocyanate

8. A final wash with 10 ml 15 mM citrate, 150 mM NaCl pH7, +0.03% NaN_3.

9. Store in 20% ethanol.

Solutions

1. 0.1M NaOAC pH4.7. >1.23g/150 ml anhydrous; adjust pH with acetic acid.
2. 3mM Sodium m-periodate: 6.42mg/10ml 0.1M Sodium acetate (above).
3. 0.1M Trisodium citrate; 50mM Sodium carbonate pH10, pH with HCl

   >> 4.41/0.795g/150 ml
4. 100mM Ascorbic acid: 0.176/10ml
5. 1M Ethanolamine pH9.0: 6.1g/100ml
6. 0.1M Glycine pH3.0: 0.376g/50ml
7. 5M Urea pH6.0: 15g/50ml
8. 3M Potassium thiocyanate: 14.6g/50ml
9. 15mM Citrate, 150mM Sodium chloride pH7:0.315g & 0.87g/100mL

All solutions were filtered sterilized with 0.22µ filters. The proper pH was achieved using HCl or NaOH.
