# Supplementary material for: Transcription Factors Bind Thousands of Active and Inactive Regions in the Drosophila Blastoderm
Source: PLoS Biol. 2008 Feb 12;6(2):e27. doi: 10.1371/journal.pbio.0060027 (PMC2235902; doi:10.1371/journal.pbio.0060027)
Supplement: Supplementary file 2 [file pbio.0060027.sg001.pdf]

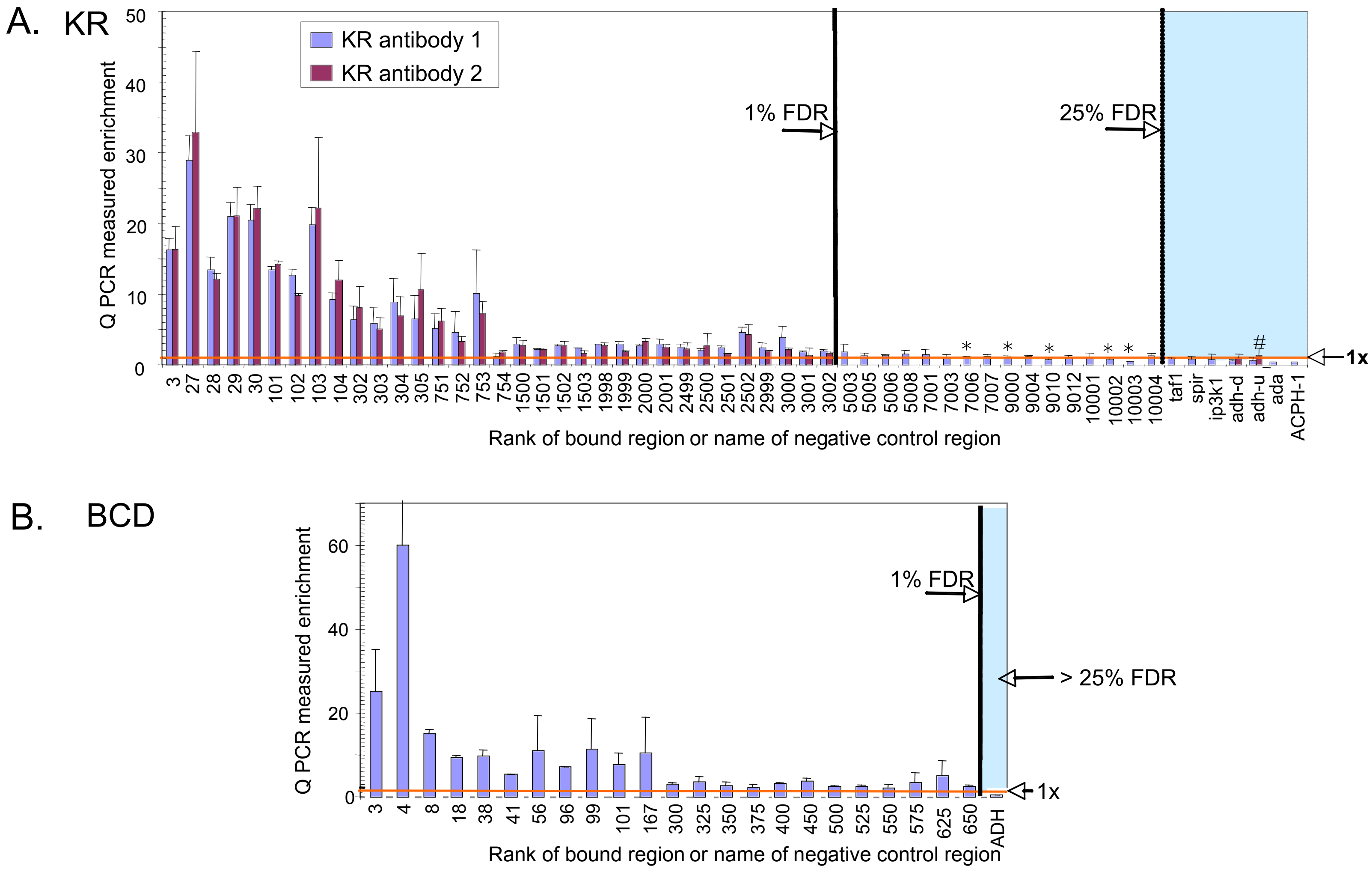
A.
KR
KR antibody 1
KR antibody 2
1% FDR
25% FDR
Q PCR measured enrichment
0
10
20
30
40
50
1x
3 27 28 29 30 101 102 103 104 302 303 304 305 751 752 753 754 1500 1501 1502 1503 1998 1999 2000 2001 2499 2500 2501 2502 2999 3000 3001 3002 5003 5005 5006 5008 7001 7003 7006 7007 9000 9004 9010 9012 10001 10002 10003 10004 taf1 spir ip3k1 adh-d adh-u ada ACPH-1
Rank of bound region or name of negative control region
B.
BCD
1% FDR
> 25% FDR
Q PCR measured enrichment
0
20
40
60
1x
3 4 8 18 38 41 56 96 99 101 167 300 325 350 375 400 450 500 525 550 575 625 650 ADH
Rank of bound region or name of negative control region

Figure S1
