# Supplementary material for: Transcription Factors Bind Thousands of Active and Inactive Regions in the Drosophila Blastoderm
Source: PLoS Biol. 2008 Feb 12;6(2):e27. doi: 10.1371/journal.pbio.0060027 (PMC2235902; doi:10.1371/journal.pbio.0060027)
Supplement: Supplementary file 3 [file pbio.0060027.sg002.pdf]

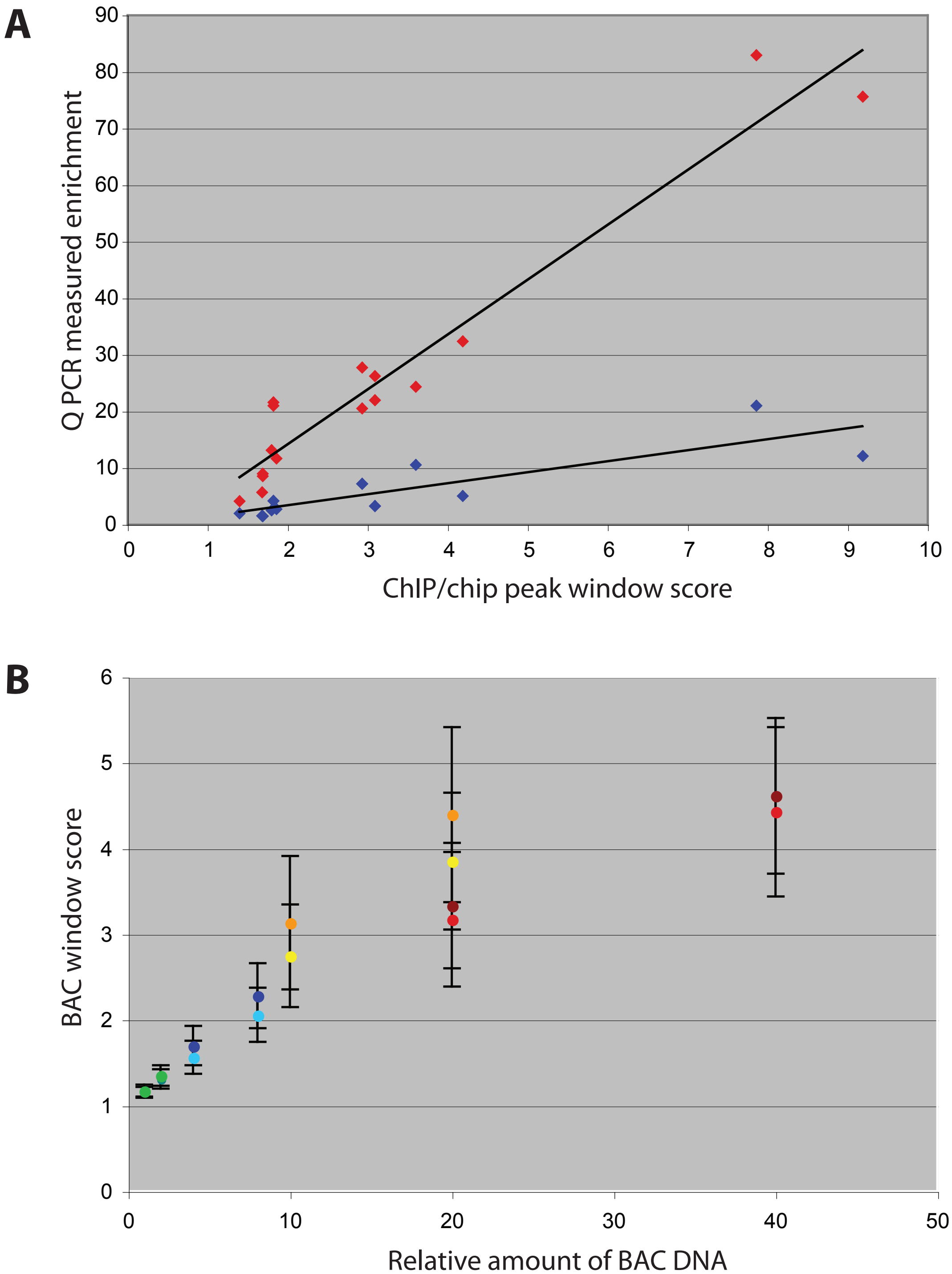
A
Q PCR measured enrichment
ChIP/chip peak window score
B
BAC window score
Relative amount of BAC DNA

Figure S2
