# Supplementary material for: Transcription Factors Bind Thousands of Active and Inactive Regions in the Drosophila Blastoderm
Source: PLoS Biol. 2008 Feb 12;6(2):e27. doi: 10.1371/journal.pbio.0060027 (PMC2235902; doi:10.1371/journal.pbio.0060027)
Supplement: Supplementary file 4 [file pbio.0060027.sg003.pdf]

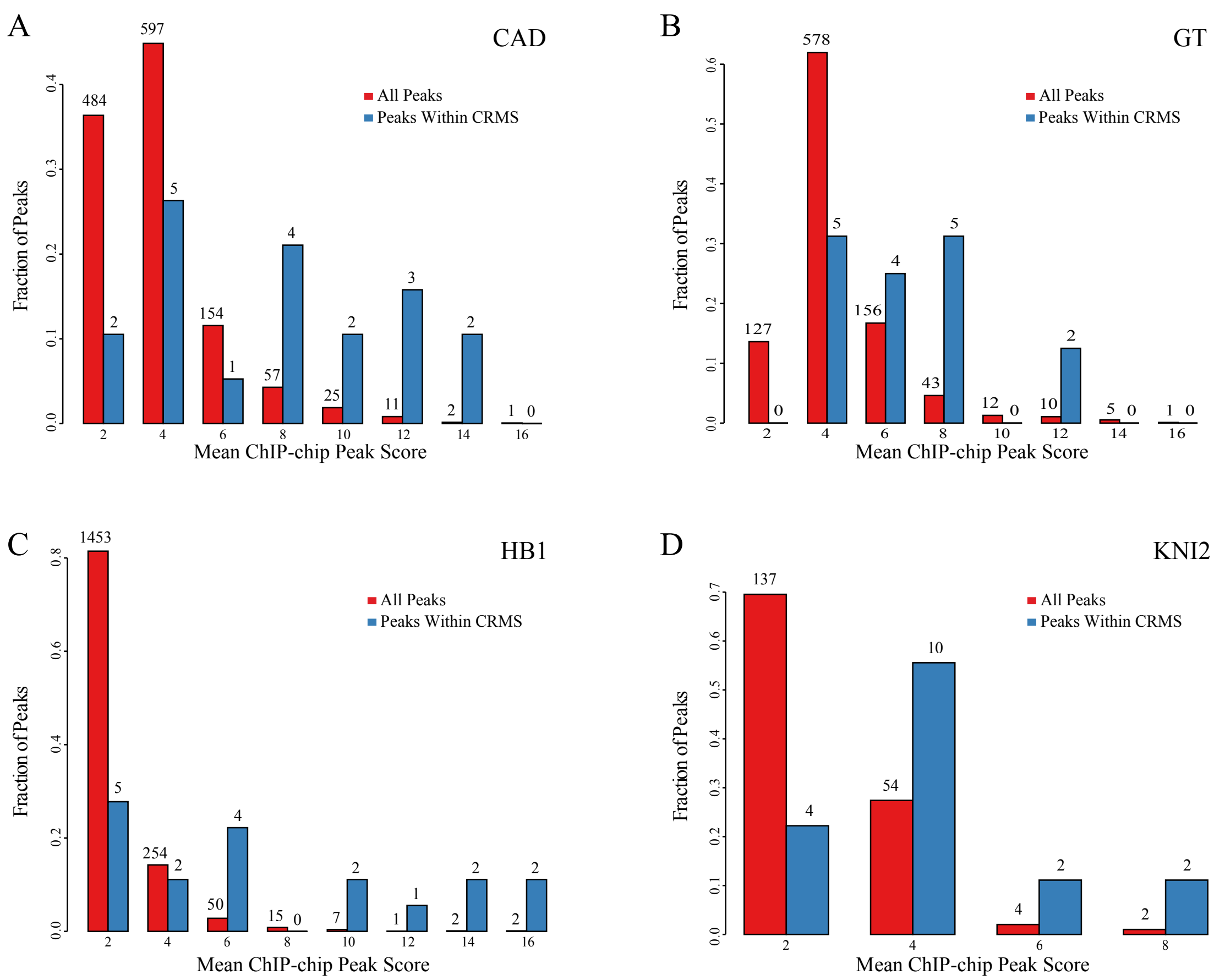
A
CAD
All Peaks
Peaks Within CRMS
Fraction of Peaks
Mean ChIP-chip Peak Score
484 2 597 5 154 1 57 4 25 2 11 3 2 2 1 0
B
GT
All Peaks
Peaks Within CRMS
Fraction of Peaks
Mean ChIP-chip Peak Score
127 0 578 5 156 4 43 5 12 0 10 2 5 0 1 0
C
HB1
All Peaks
Peaks Within CRMS
Fraction of Peaks
Mean ChIP-chip Peak Score
1453 5 254 2 50 4 15 0 7 2 1 1 2 2 2 2
D
KNI2
All Peaks
Peaks Within CRMS
Fraction of Peaks
Mean ChIP-chip Peak Score
137 4 54 10 4 2 2 2

Figure S3
