# Supplementary material for: Transcription Factors Bind Thousands of Active and Inactive Regions in the Drosophila Blastoderm
Source: PLoS Biol. 2008 Feb 12;6(2):e27. doi: 10.1371/journal.pbio.0060027 (PMC2235902; doi:10.1371/journal.pbio.0060027)
Supplement: Supplementary file 5 [file pbio.0060027.sg004.pdf]

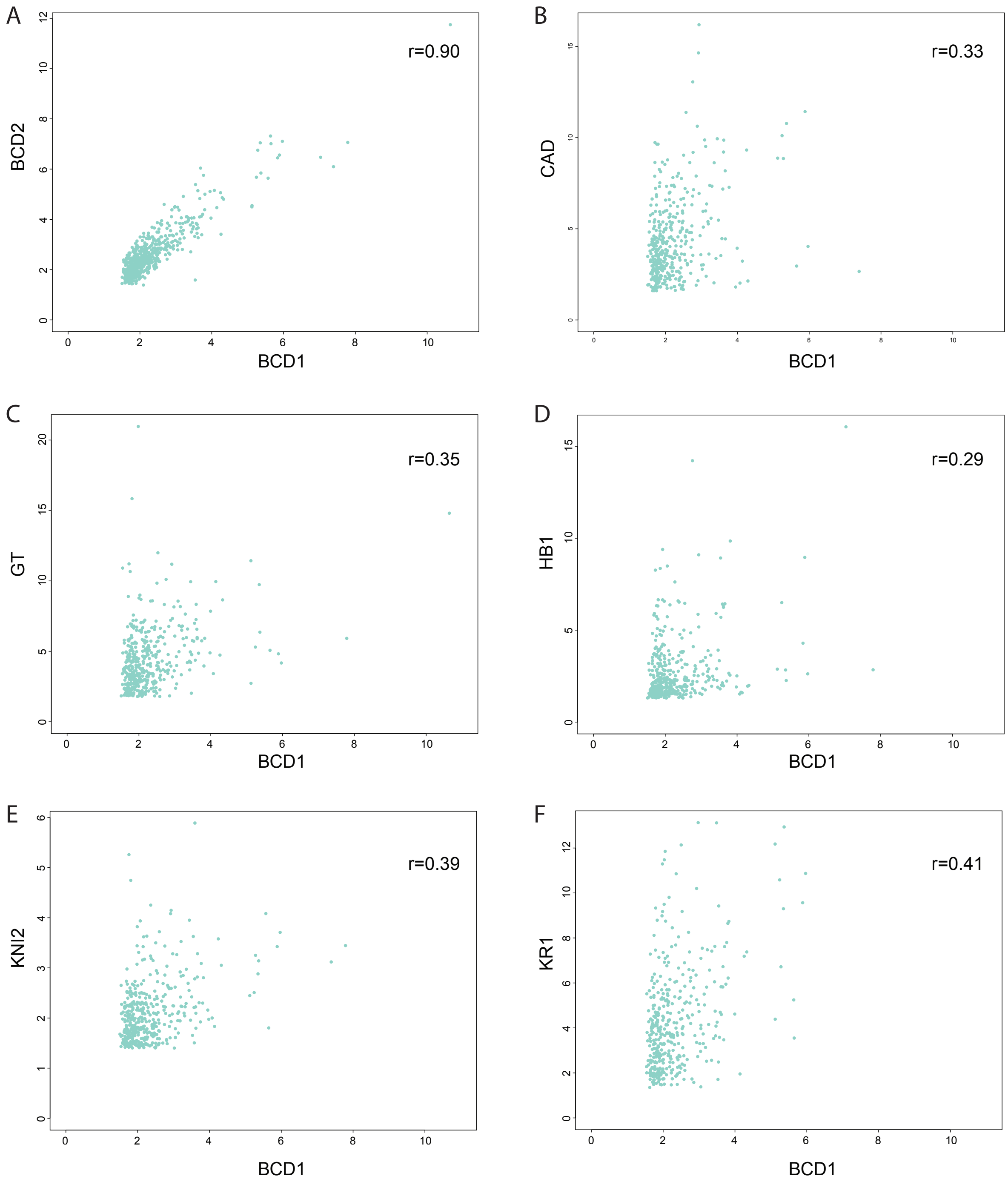
A
BCD2
BCD1
r=0.90
B
CAD
BCD1
r=0.33
C
GT
BCD1
r=0.35
D
HB1
BCD1
r=0.29
E
KNI2
BCD1
r=0.39
F
KR1
BCD1
r=0.41

Figure S4-1

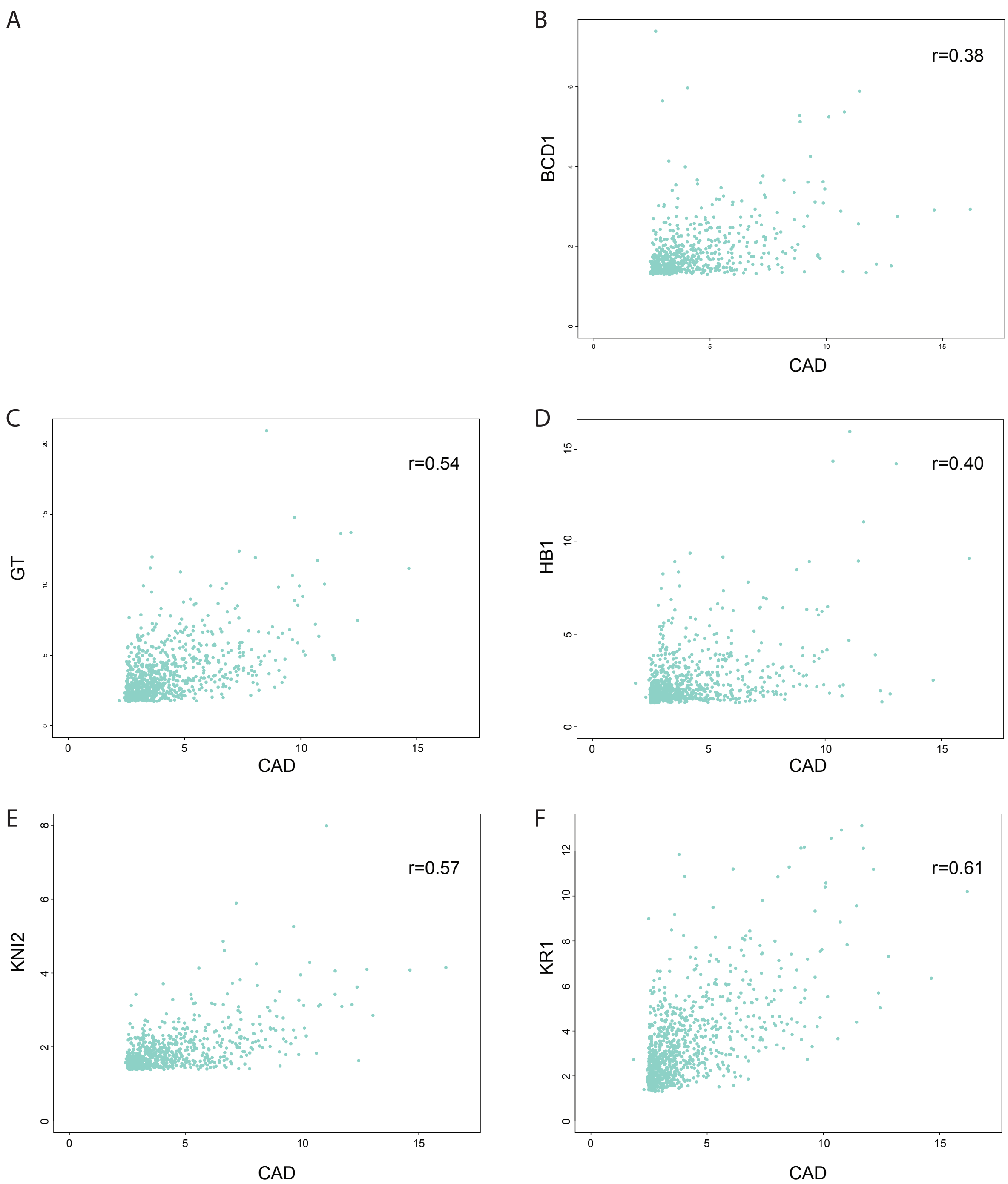
A
B
r=0.38
BCD1
CAD
C
r=0.54
GT
CAD
D
r=0.40
HB1
CAD
E
r=0.57
KNI2
CAD
F
r=0.61
KR1
CAD

Figure S4-2

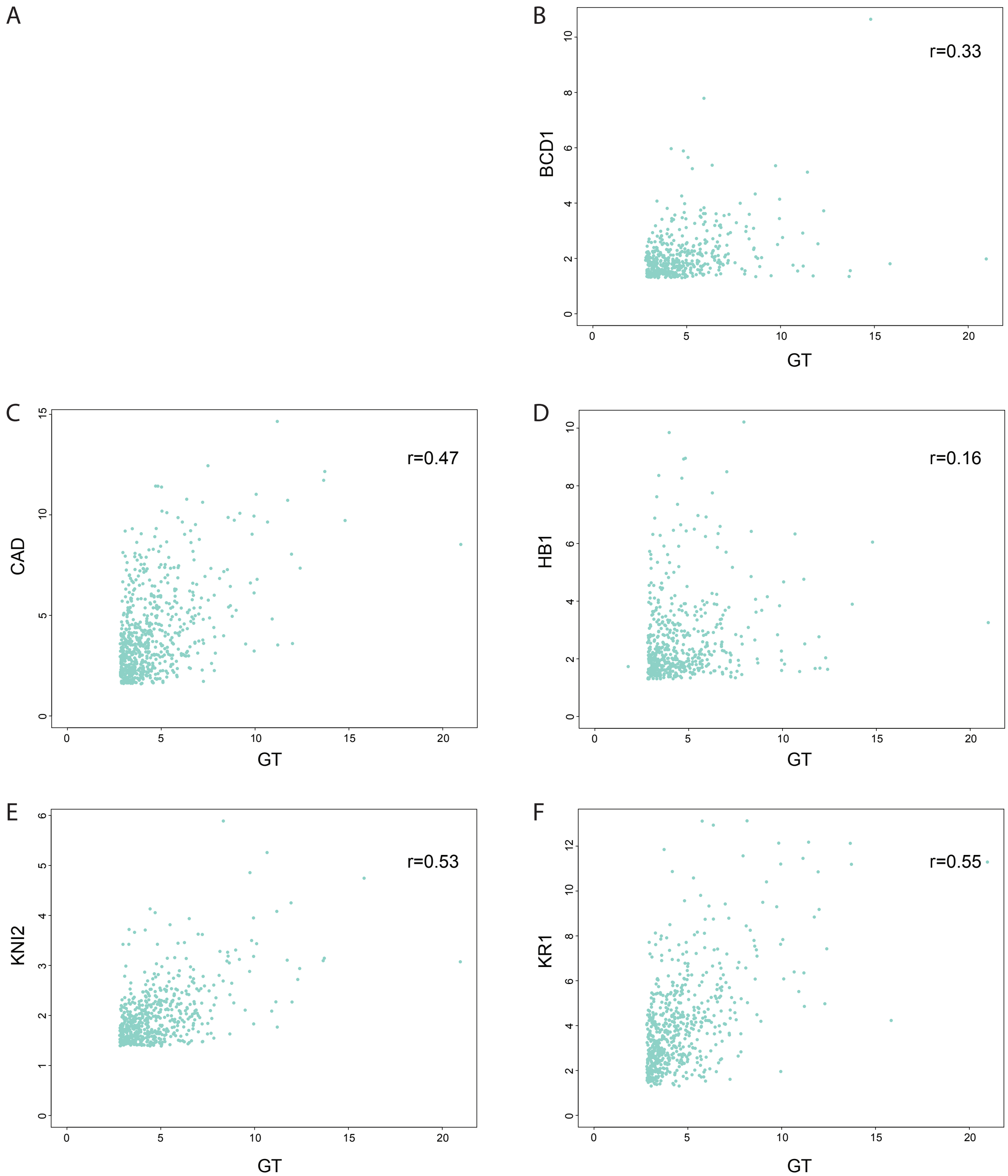
A
B
r=0.33
BCD1
GT
C
r=0.47
CAD
GT
D
r=0.16
HB1
GT
E
r=0.53
KNI2
GT
F
r=0.55
KR1
GT

Figure S4-3

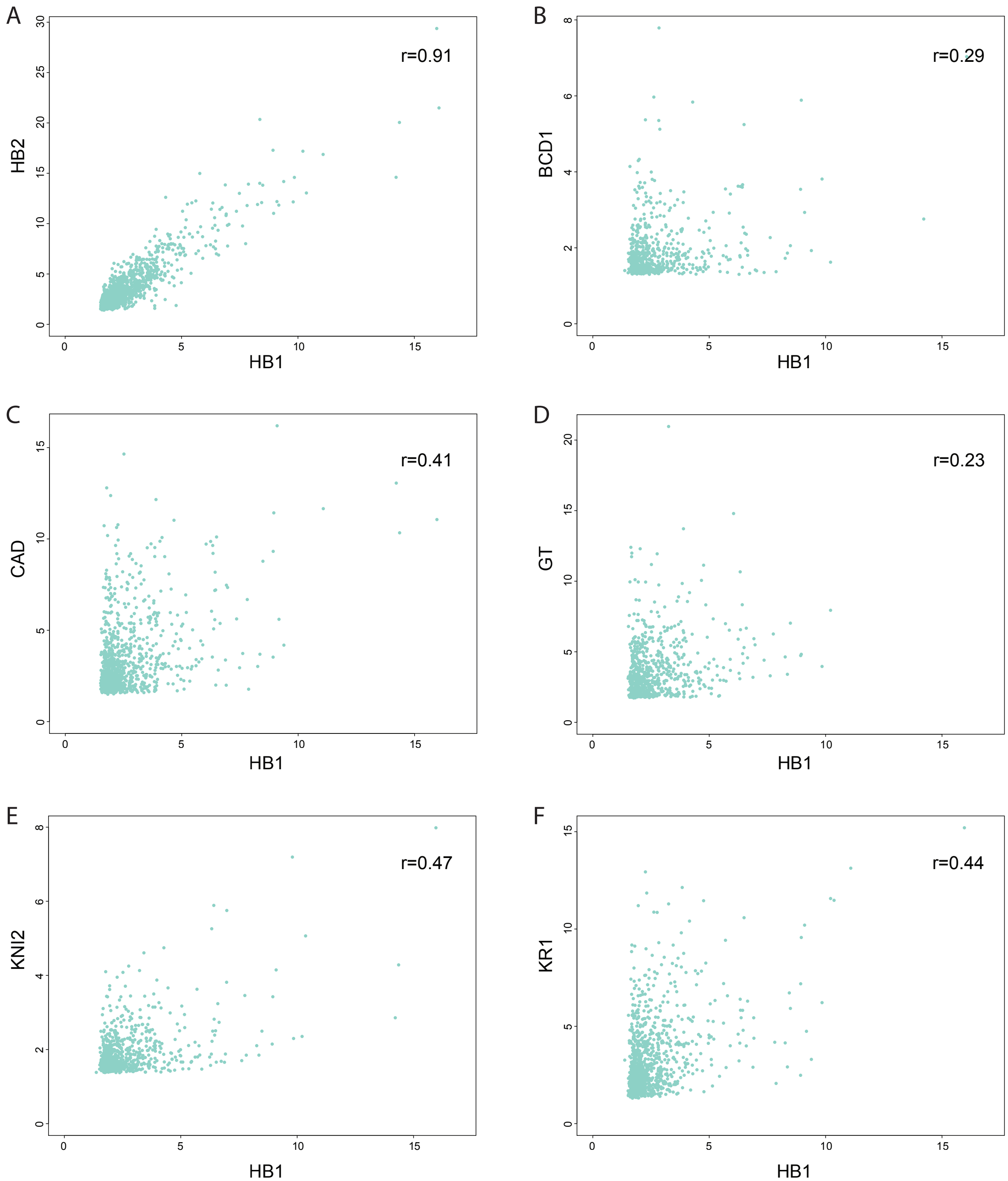
A
r=0.91
HB2
HB1
B
r=0.29
BCD1
HB1
C
r=0.41
CAD
HB1
D
r=0.23
GT
HB1
E
r=0.47
KNI2
HB1
F
r=0.44
KR1
HB1

Figure S4-4

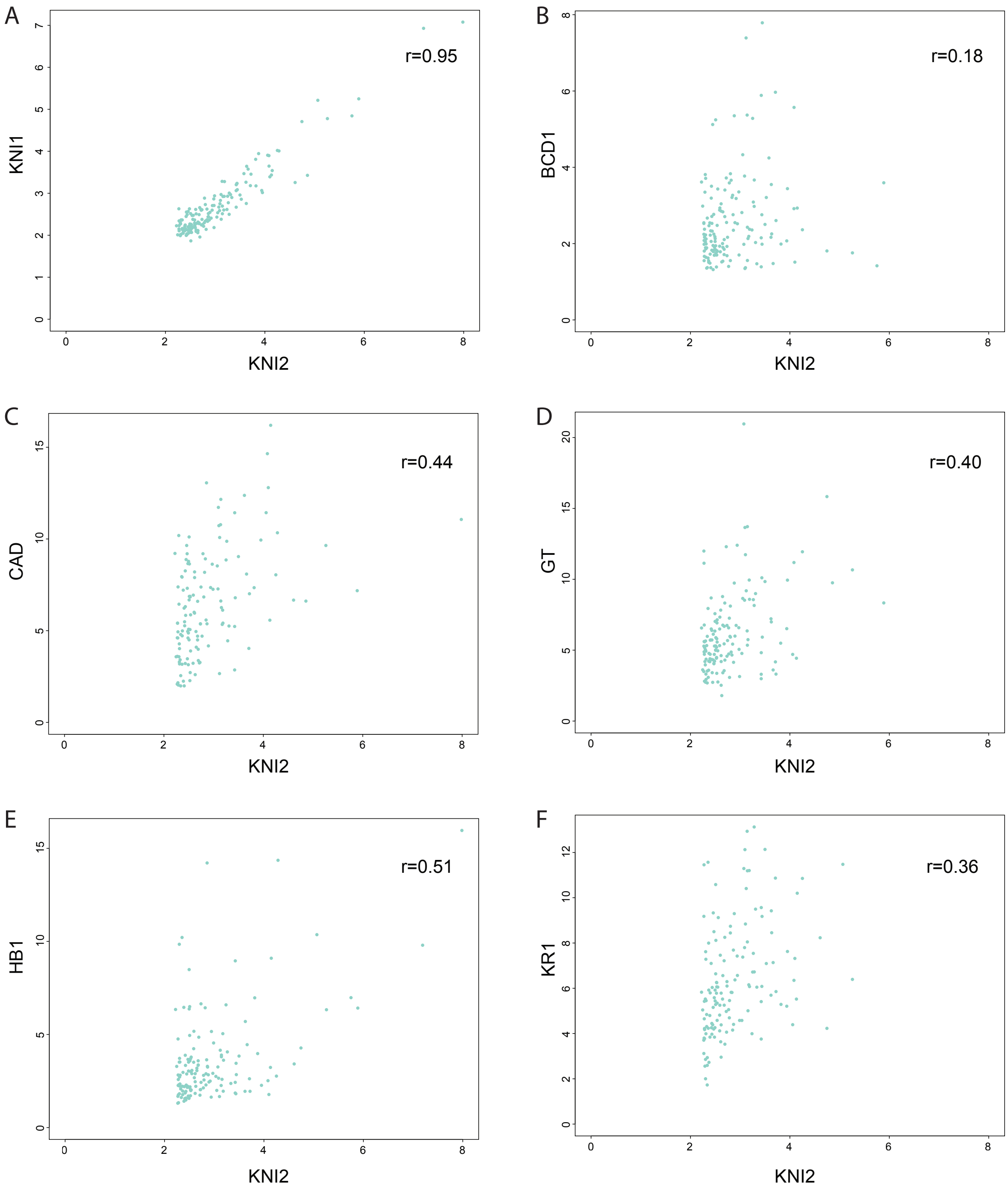
A
KNI1
KNI2
r=0.95
B
BCD1
KNI2
r=0.18
C
CAD
KNI2
r=0.44
D
GT
KNI2
r=0.40
E
HB1
KNI2
r=0.51
F
KR1
KNI2
r=0.36

Figure S4-5
