# Supplementary material for: Transcription Factors Bind Thousands of Active and Inactive Regions in the Drosophila Blastoderm
Source: PLoS Biol. 2008 Feb 12;6(2):e27. doi: 10.1371/journal.pbio.0060027 (PMC2235902; doi:10.1371/journal.pbio.0060027)
Supplement: Supplementary file 6 [file pbio.0060027.sg005.pdf]

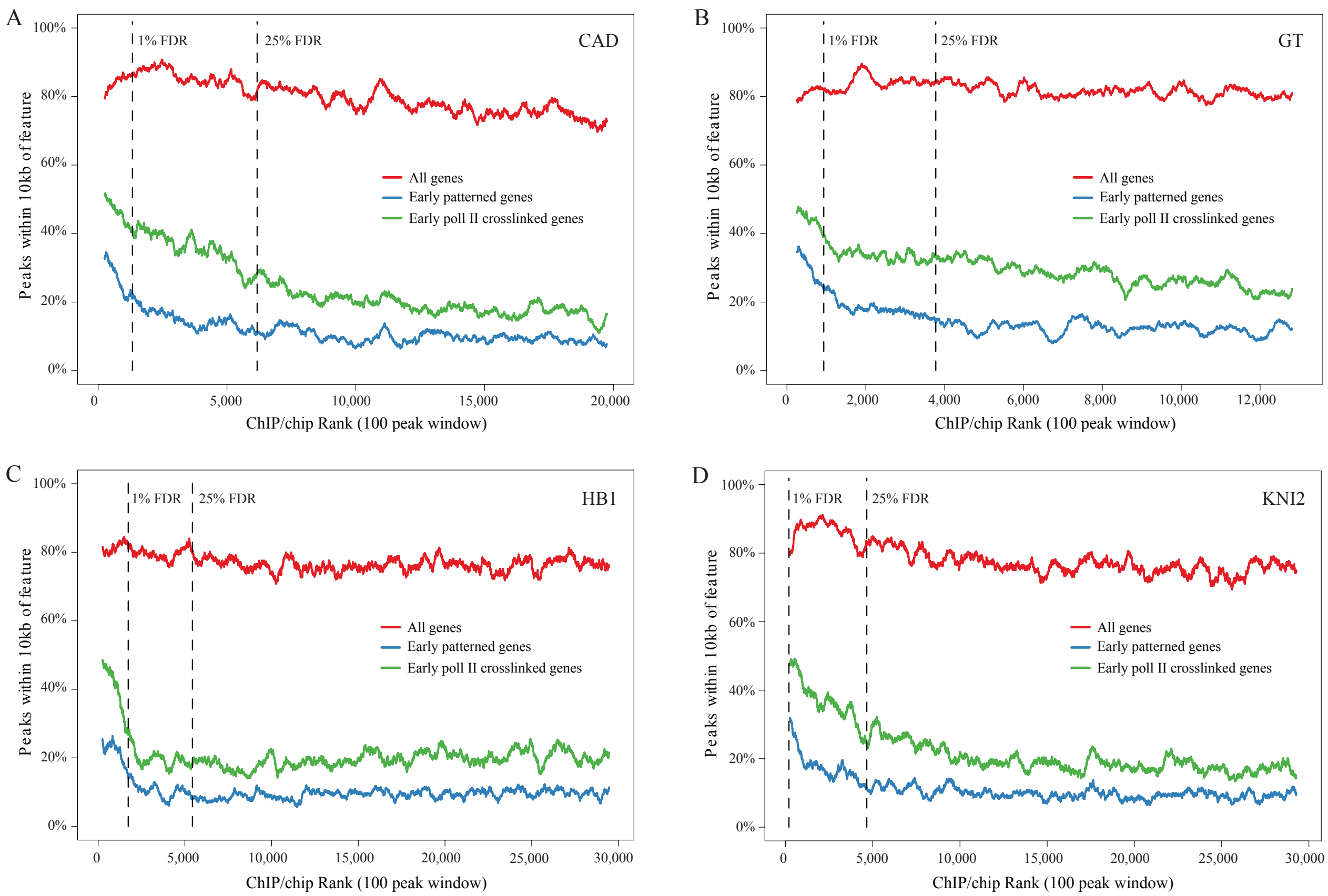
A
100%
80%
60%
40%
20%
0%
1% FDR
25% FDR
CAD
All genes
Early patterned genes
Early poll II crosslinked genes
Peaks within 10kb of feature
0
5,000
10,000
15,000
20,000
ChIP/chip Rank (100 peak window)
B
1% FDR
25% FDR
GT
All genes
Early patterned genes
Early poll II crosslinked genes
Peaks within 10kb of feature
0
2,000
4,000
6,000
8,000
10,000
12,000
ChIP/chip Rank (100 peak window)
C
1% FDR
25% FDR
HB1
All genes
Early patterned genes
Early poll II crosslinked genes
Peaks within 10kb of feature
0
5,000
10,000
15,000
20,000
25,000
30,000
ChIP/chip Rank (100 peak window)
D
1% FDR
25% FDR
KNI2
All genes
Early patterned genes
Early poll II crosslinked genes
Peaks within 10kb of feature
0
5,000
10,000
15,000
20,000
25,000
30,000
ChIP/chip Rank (100 peak window)

Figure S5
