# Supplementary material for: Transcription Factors Bind Thousands of Active and Inactive Regions in the Drosophila Blastoderm
Source: PLoS Biol. 2008 Feb 12;6(2):e27. doi: 10.1371/journal.pbio.0060027 (PMC2235902; doi:10.1371/journal.pbio.0060027)
Supplement: Supplementary file 7 [file pbio.0060027.sg006.pdf]

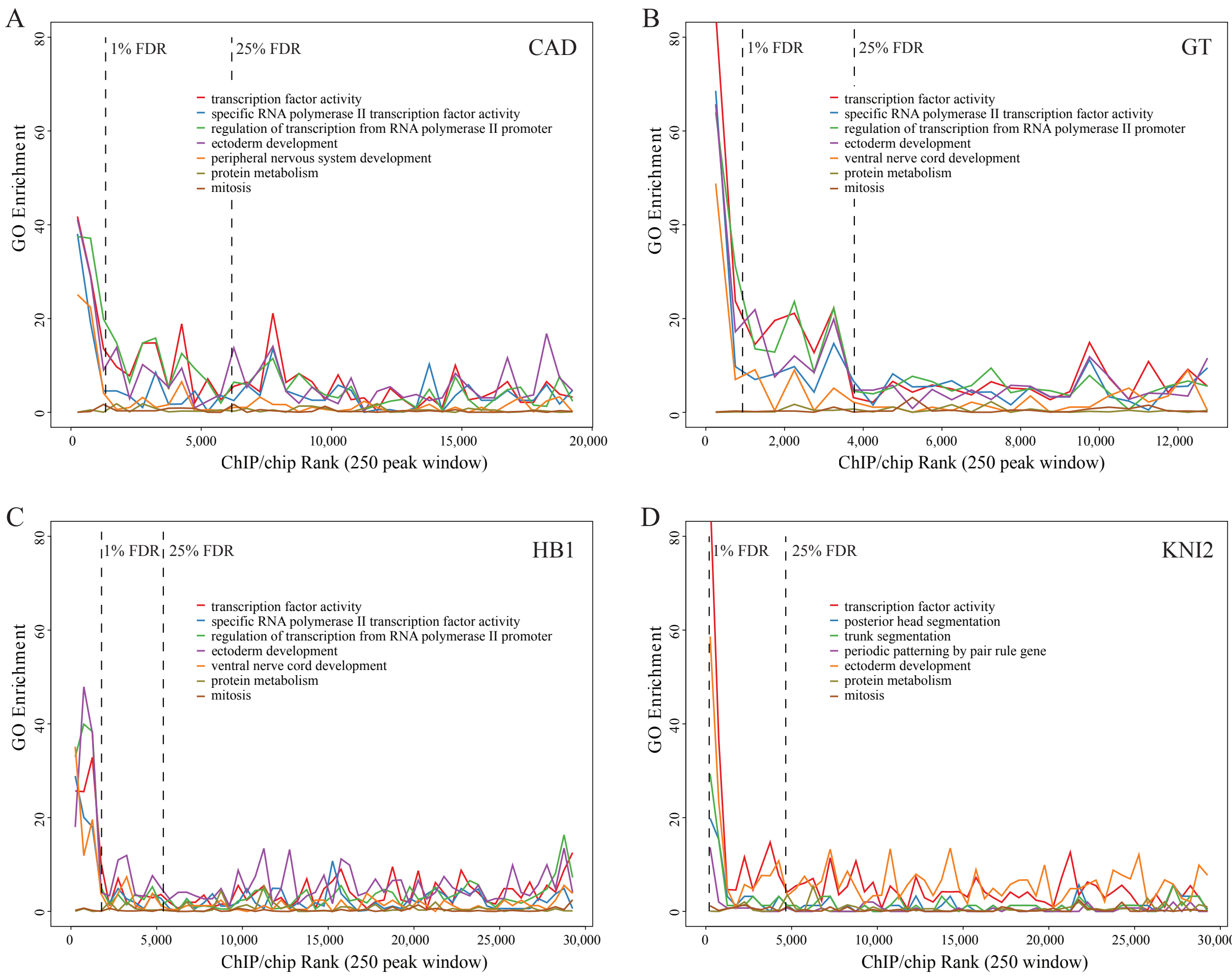
A
CAD
1% FDR
25% FDR
transcription factor activity
specific RNA polymerase II transcription factor activity
regulation of transcription from RNA polymerase II promoter
ectoderm development
peripheral nervous system development
protein metabolism
mitosis
GO Enrichment
ChIP/chip Rank (250 peak window)
B
GT
1% FDR
25% FDR
transcription factor activity
specific RNA polymerase II transcription factor activity
regulation of transcription from RNA polymerase II promoter
ectoderm development
ventral nerve cord development
protein metabolism
mitosis
GO Enrichment
ChIP/chip Rank (250 peak window)
C
HB1
1% FDR
25% FDR
transcription factor activity
specific RNA polymerase II transcription factor activity
regulation of transcription from RNA polymerase II promoter
ectoderm development
ventral nerve cord development
protein metabolism
mitosis
GO Enrichment
ChIP/chip Rank (250 peak window)
D
KNI2
1% FDR
25% FDR
transcription factor activity
posterior head segmentation
trunk segmentation
periodic patterning by pair rule gene
ectoderm development
protein metabolism
mitosis
GO Enrichment
ChIP/chip Rank (250 window)

Figure S6
