# Supplementary material for: Transcription Factors Bind Thousands of Active and Inactive Regions in the Drosophila Blastoderm
Source: PLoS Biol. 2008 Feb 12;6(2):e27. doi: 10.1371/journal.pbio.0060027 (PMC2235902; doi:10.1371/journal.pbio.0060027)
Supplement: Supplementary file 8 [file pbio.0060027.sg007.pdf]

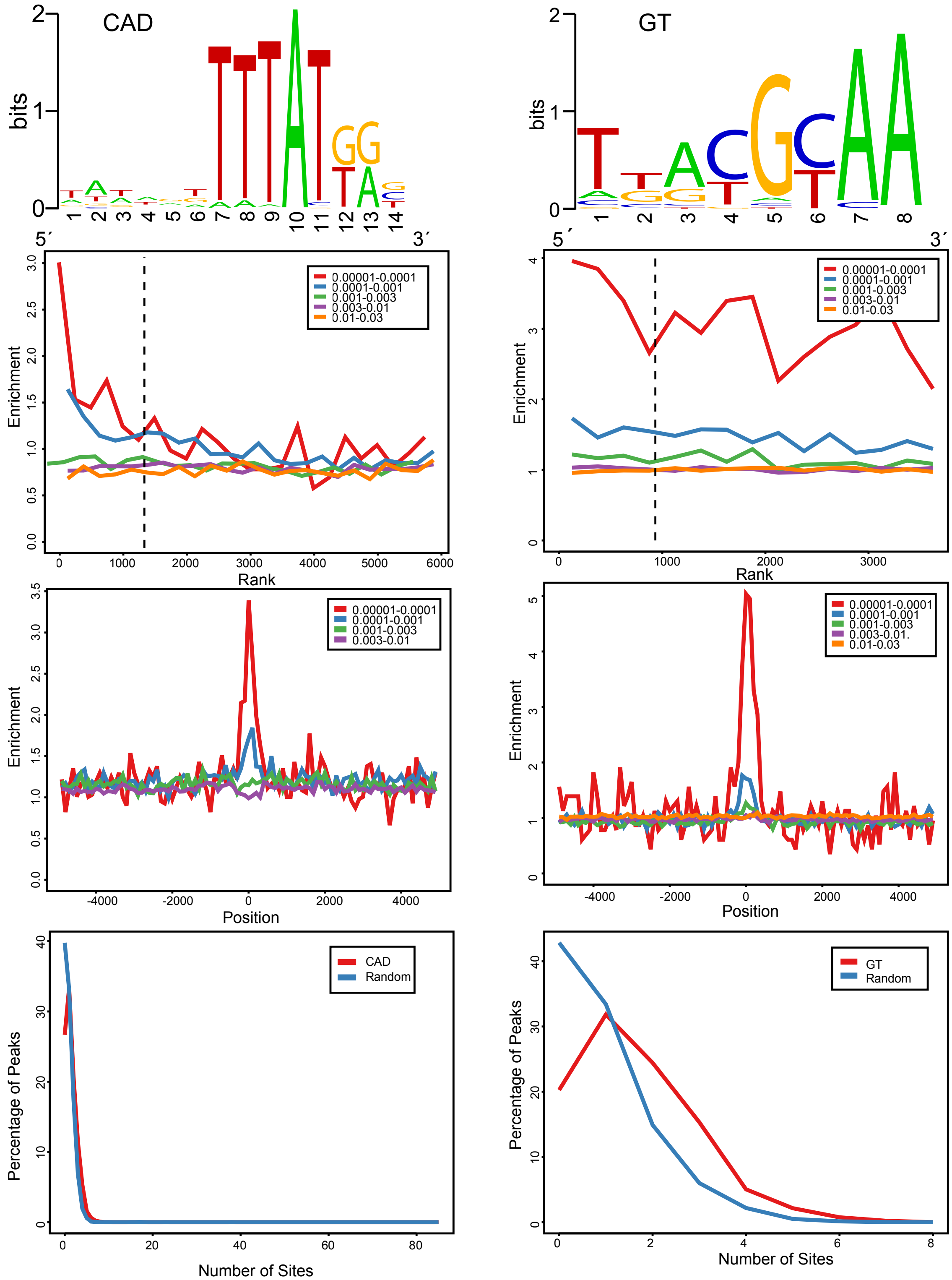

CAD
GT
bits
5´
3´
Enrichment
Rank
Position
Percentage of Peaks
Number of Sites
0.00001-0.0001
0.0001-0.001
0.001-0.003
0.003-0.01
0.01-0.03
CAD
GT
Random

Figure S7-1

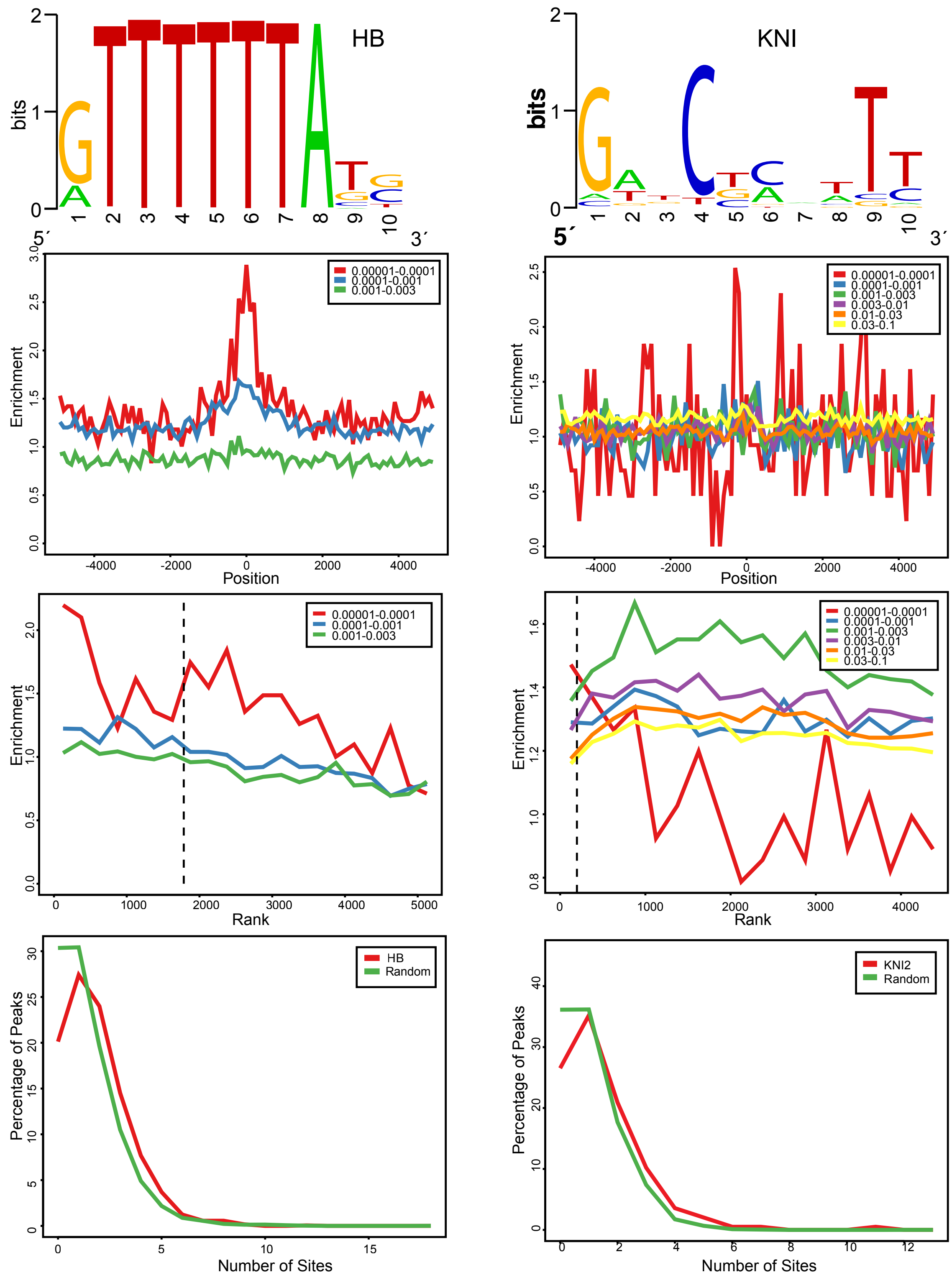

HB
KNI
bits
5´
3´
Enrichment
Position
Rank
Percentage of Peaks
Number of Sites
0.00001-0.0001
0.0001-0.001
0.001-0.003
0.003-0.01
0.01-0.03
0.03-0.1
HB
Random
KNI2
Random

Figure S7-2
