# Supplementary material for: Transcription Factors Bind Thousands of Active and Inactive Regions in the Drosophila Blastoderm
Source: PLoS Biol. 2008 Feb 12;6(2):e27. doi: 10.1371/journal.pbio.0060027 (PMC2235902; doi:10.1371/journal.pbio.0060027)
Supplement: Supplementary file 10 [file pbio.0060027.sg009.pdf]

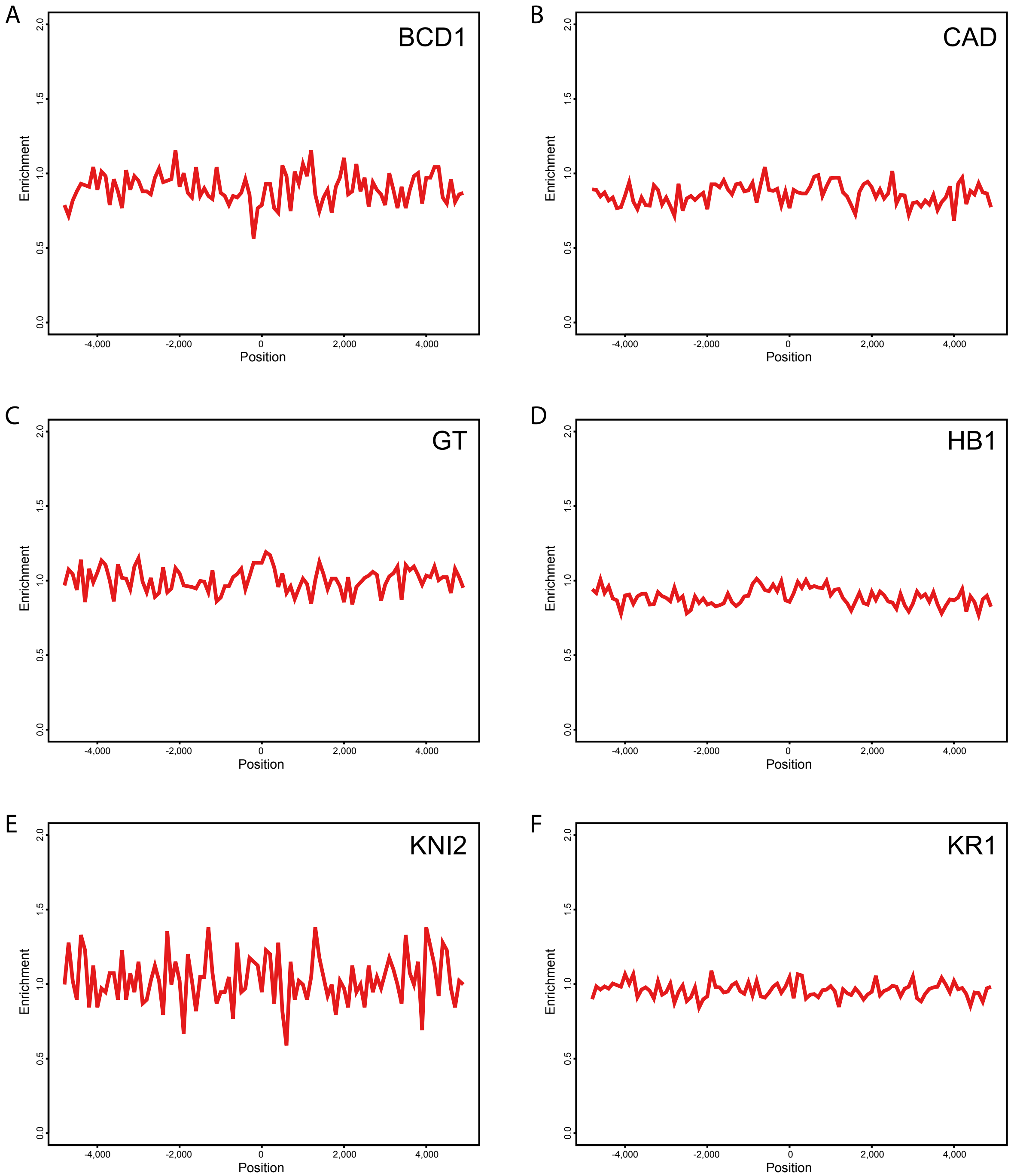
A
BCD1
B
CAD
C
GT
D
HB1
E
KNI2
F
KR1
Enrichment
0.0
0.5
1.0
1.5
2.0
-4,000
-2,000
0
2,000
4,000
Position

Figure S9
