# Supplementary material for: Transcription Factors Bind Thousands of Active and Inactive Regions in the Drosophila Blastoderm
Source: PLoS Biol. 2008 Feb 12;6(2):e27. doi: 10.1371/journal.pbio.0060027 (PMC2235902; doi:10.1371/journal.pbio.0060027)
Supplement: Supplementary file 11 [file pbio.0060027.sg010.pdf]

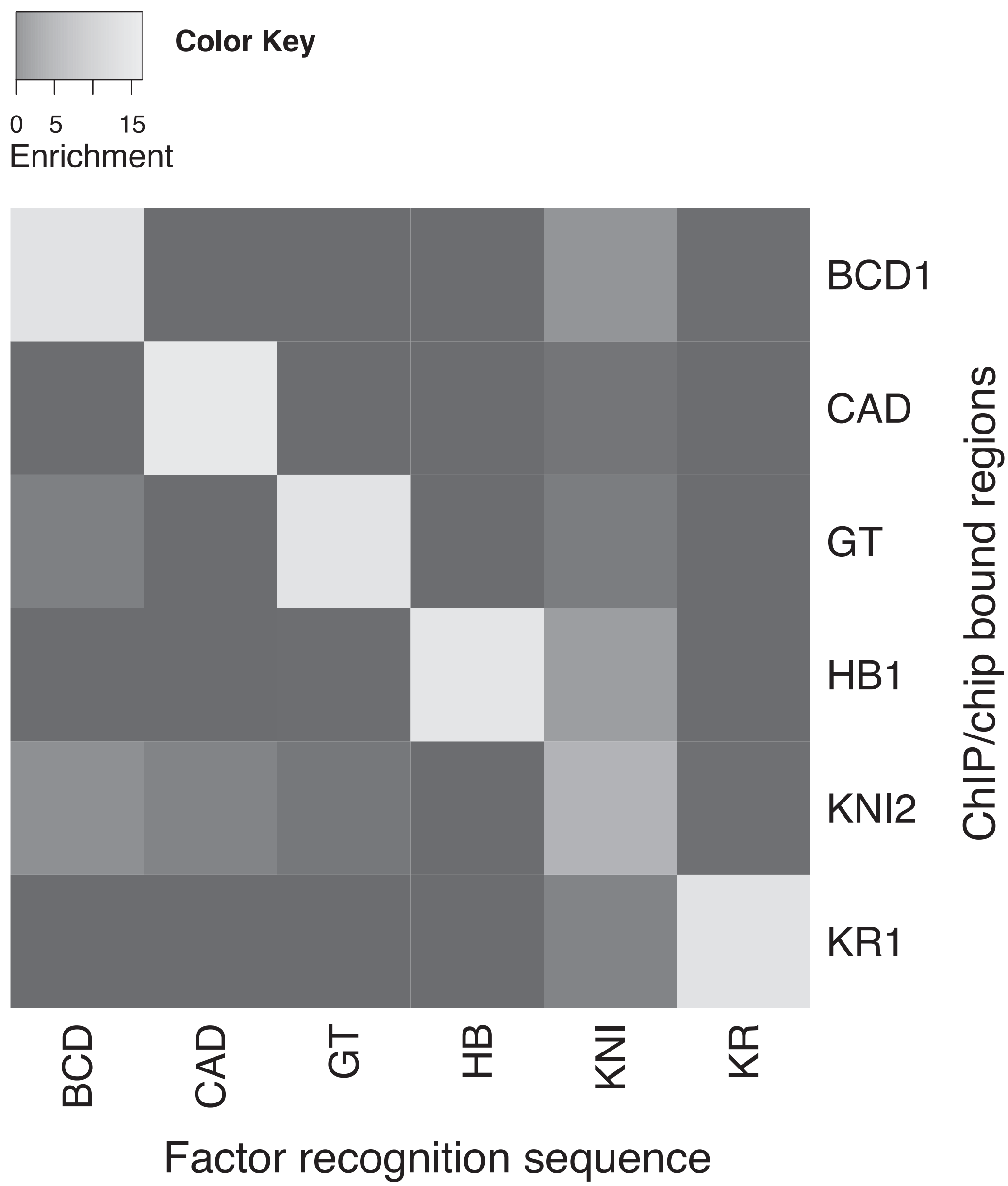

Color Key
0 5 15
Enrichment
BCD1
CAD
GT
HB1
KNI2
KR1
ChIP/chip bound regions
BCD
CAD
GT
HB
KNI
KR
Factor recognition sequence

Figure S10
