# Supplementary material for: Transcription Factors Bind Thousands of Active and Inactive Regions in the Drosophila Blastoderm
Source: PLoS Biol. 2008 Feb 12;6(2):e27. doi: 10.1371/journal.pbio.0060027 (PMC2235902; doi:10.1371/journal.pbio.0060027)
Supplement: Supplementary file 12 [file pbio.0060027.sg011.pdf]

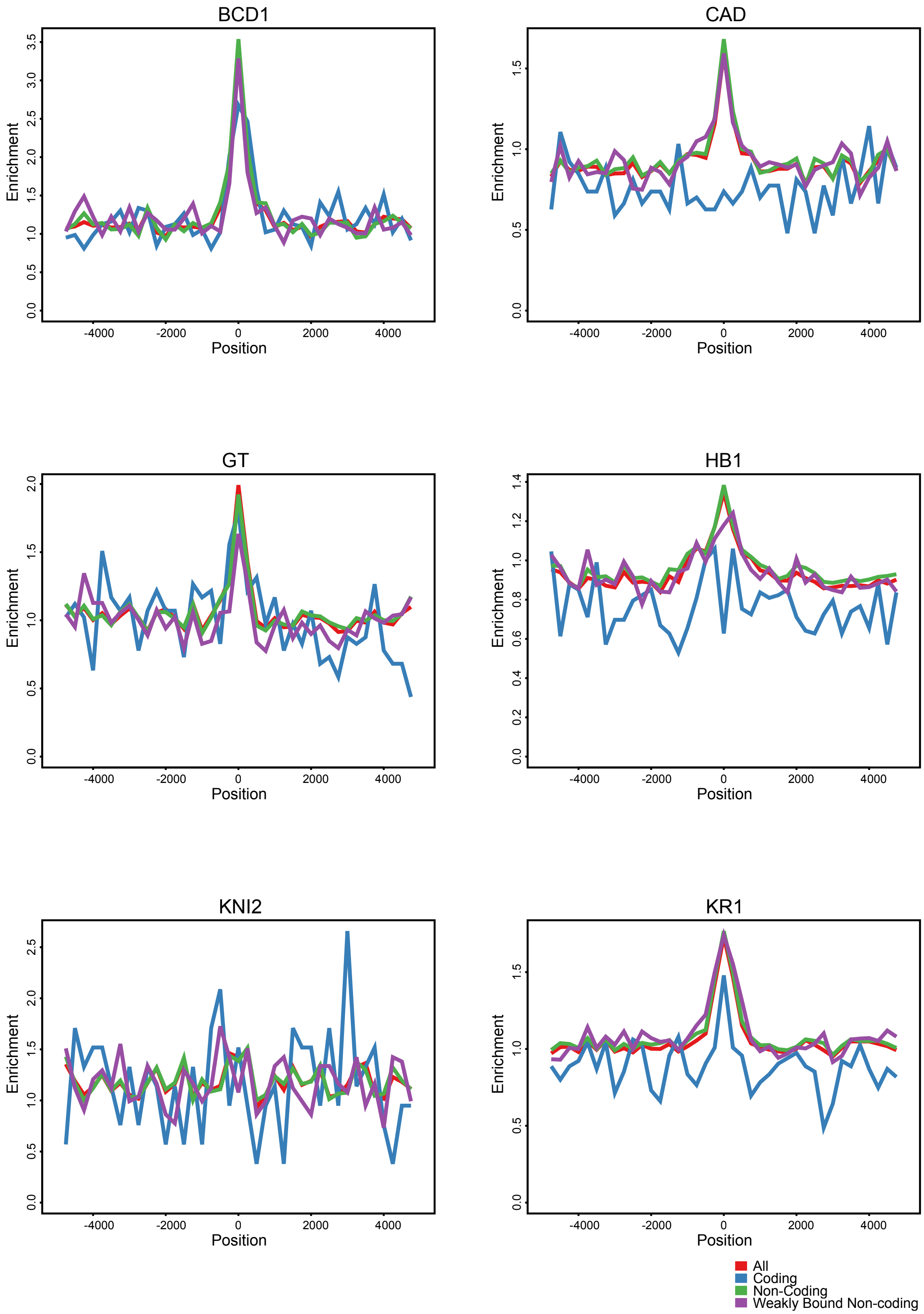
BCD1
CAD
GT
HB1
KNI2
KR1
Enrichment
Position
-4000
-2000
0
2000
4000
All
Coding
Non-Coding
Weakly Bound Non-coding

Figure S11
