# Supplementary material for: Transcription Factors Bind Thousands of Active and Inactive Regions in the Drosophila Blastoderm
Source: PLoS Biol. 2008 Feb 12;6(2):e27. doi: 10.1371/journal.pbio.0060027 (PMC2235902; doi:10.1371/journal.pbio.0060027)
Supplement: Supplementary file 13 [file pbio.0060027.sg012.pdf]

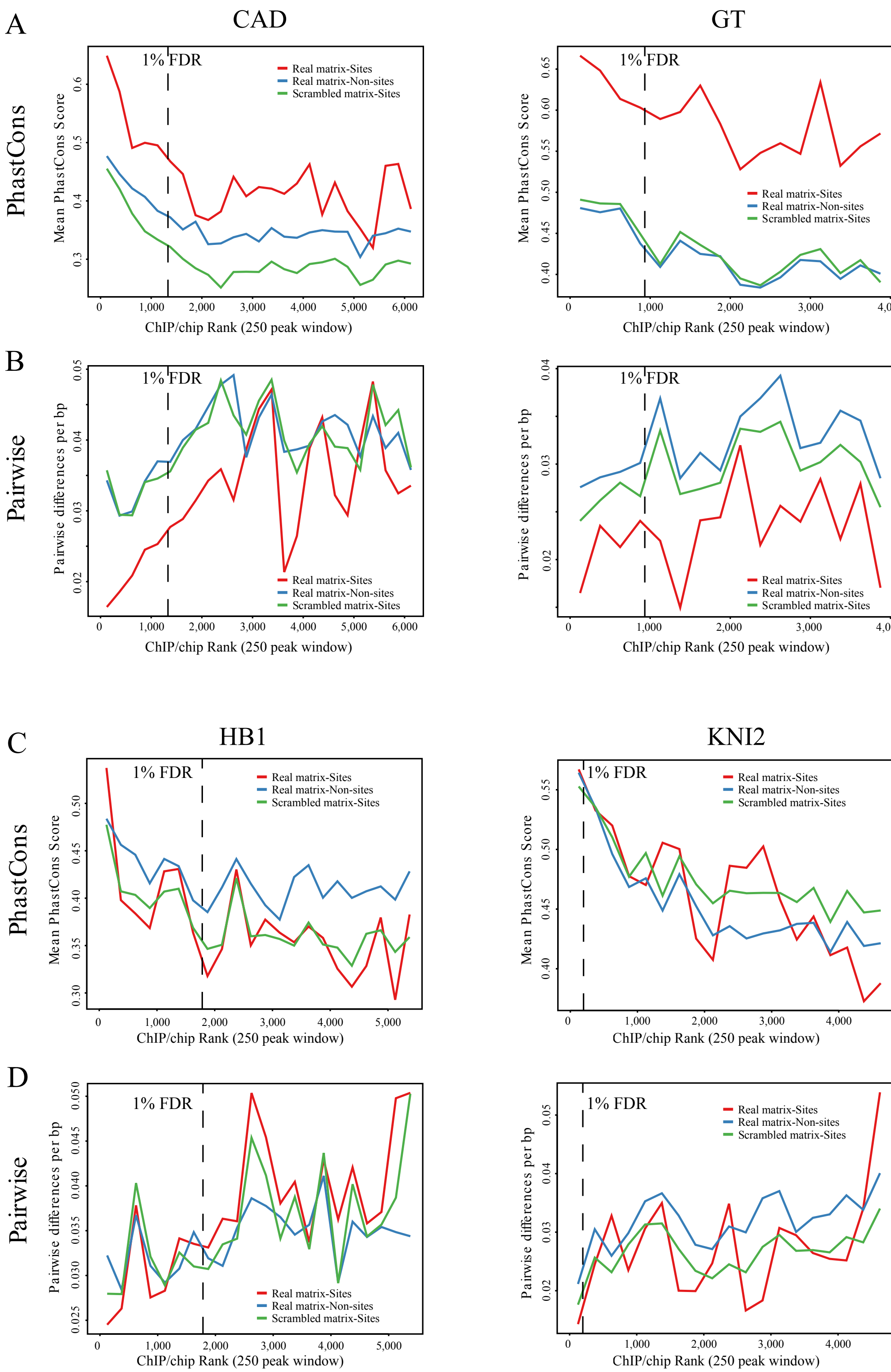
A
CAD
GT
PhastCons
Mean PhastCons Score
1% FDR
Real matrix-Sites
Real matrix-Non-sites
Scrambled matrix-Sites
ChIP/chip Rank (250 peak window)
B
Pairwise
Pairwise differences per bp
C
HB1
KNI2
D

Figure S12
